# Supplementary figures and images for: Recent range expansion and lineage idiosyncratic population structure of Liodessus diving beetles in the high Andes (Coleoptera: Dytiscidae, Bidessini)
Source: PLoS One. 2024 Dec 20;19(12):e0308683. doi: 10.1371/journal.pone.0308683 (PMC11661648; doi:10.1371/journal.pone.0308683)

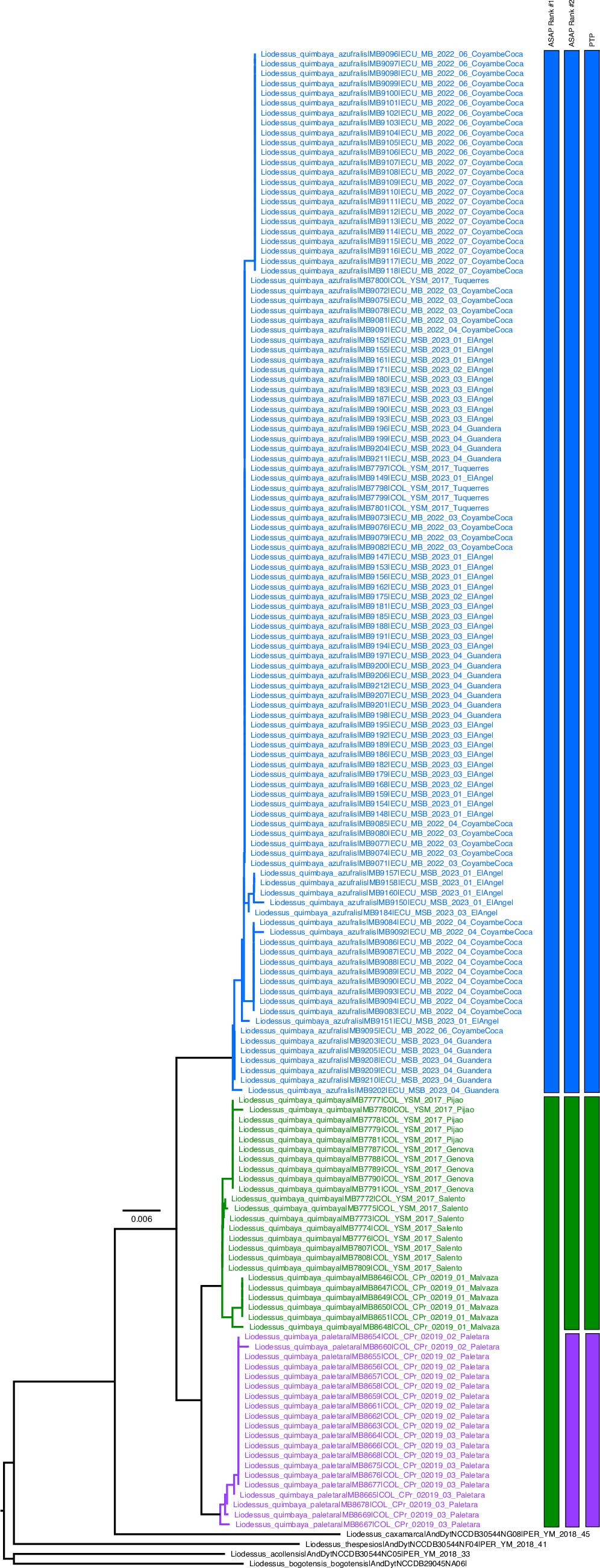

Supplement: S1 Fig — (TIF) [file pone.0308683.s001.tif]

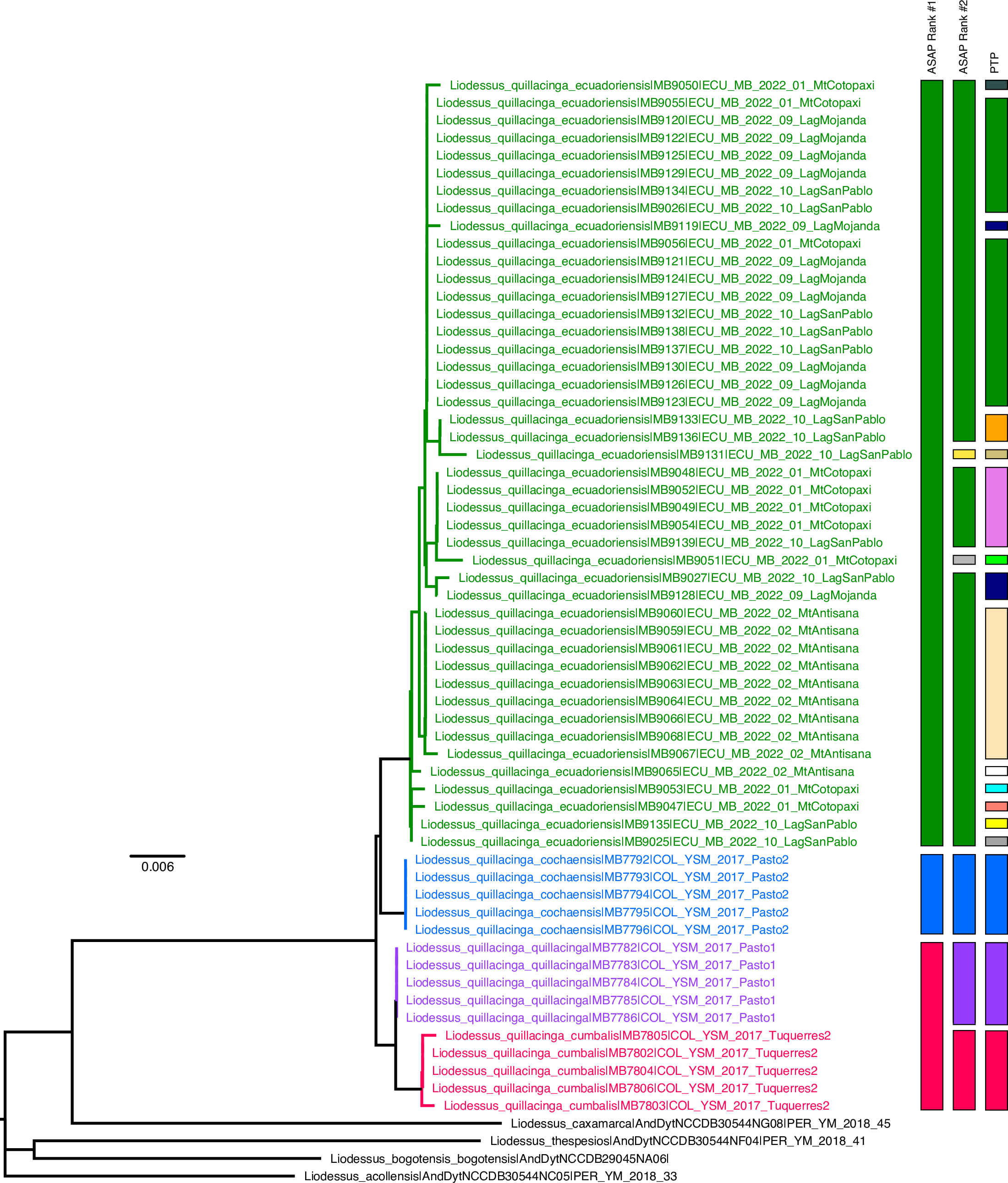

Supplement: S2 Fig — (TIF) [file pone.0308683.s002.tif]
