# Supplementary material for: Recent range expansion and lineage idiosyncratic population structure of Liodessus diving beetles in the high Andes (Coleoptera: Dytiscidae, Bidessini)
Source: PLoS One. 2024 Dec 20;19(12):e0308683. doi: 10.1371/journal.pone.0308683 (PMC11661648; doi:10.1371/journal.pone.0308683)

PLOS ONE

Recent range expansion and lineage idiosyncratic population structure of Liodessus diving beetles in the high Andes (Coleoptera: Dytiscidae, Bidessini)

--Manuscript Draft--

**Manuscript Number:**	PONE-D-24-13211
**Article Type:**	Research Article
**Full Title:**	Recent range expansion and lineage idiosyncratic population structure of Liodessus diving beetles in the high Andes (Coleoptera: Dytiscidae, Bidessini)
**Short Title:**	Recent range expasion and lineage idiosyncratic population structure of Liodessus in the high Andes
**Corresponding Author:**	Adrián Villastrigo<br>Zoologische Staatssammlung Munchen<br>Munich, GERMANY
**Keywords:**	Colombia, Ecuador, Dytiscidae, Andes, Liodessus, new subspecies
**Abstract:**	Here, we review the taxonomy and population structure of diving beetles in the genus Liodessus Guignot, 1939 from the high Andes of southern Colombia and Ecuador. Liodessus quillacinga ecuadoriensis ssp. nov. is described from several localities in Ecuador (Type locality: Otavalo, Laguna San Pablo). Liodessus quimbaya azufralis Megna, Hendrich & Balke, 2019 stat. nov. is now regarded as a subspecies of Liodessus quimbaya Megna, Hendrich & Balke, 2019 based on new morphological and genetical data. Liodessus quimbaya paletara ssp. nov. is described from the Paletará Valley (Colombia: Cauca).
**Order of Authors:**	Michael Balke
	Tobias Mainda
	Katja Neven
	Lars Hendrich
	Michael Steven Basantes
	Carlos Prieto
	Adrián Villastrigo
**Additional Information:**	
**Question**	**Response**
**Financial Disclosure**<br><br>Enter a financial disclosure statement that describes the sources of funding for the work included in this submission. Review the submission guidelines for detailed requirements. View published research articles from *PLOS ONE* for specific examples.<br><br>This statement is required for submission and **will appear in the published article** if the submission is accepted. Please make sure it is accurate.	Yes

Powered by Editorial Manager® and ProduXion Manager® from Aries Systems Corporation

**Funded studies**<br>Enter a statement with the following details:<br>• Initials of the authors who received each award<br>• Grant numbers awarded to each author<br>• The full name of each funder<br>• URL of each funder website<br>• Did the sponsors or funders play any role in the study design, data collection and analysis, decision to publish, or preparation of the manuscript?<br><br>Did you receive funding for this work?	
Please add funding details.<br>as follow-up to "**Financial Disclosure**<br><br>Enter a financial disclosure statement that describes the sources of funding for the work included in this submission. Review the submission guidelines for detailed requirements. View published research articles from *PLOS ONE* for specific examples.<br><br>This statement is required for submission and **will appear in the published article** if the submission is accepted. Please make sure it is accurate.<br><br>**Funded studies**<br>Enter a statement with the following details:<br>• Initials of the authors who received each award<br>• Grant numbers awarded to each author<br>• The full name of each funder<br>• URL of each funder website<br>• Did the sponsors or funders play any role in the study design, data collection and analysis, decision to publish, or preparation of the manuscript?<br><br>Did you receive funding for this work?"	Michael Balke acknowledges support from the Deutsche Forschungsgemeinschaft (BA2152/27-1), project number: 496550039. In the initial phases of this project, Adrián Villastrigo was funded by the Alexander von Humboldt Foundation through a Humboldt Research Fellowship and by the Carl Friedrich von Siemens Foundation at SNSB-ZSM, and this stipend is greatly acknowledged here.<br><br>Michael Balke acknowledges support from the EU SYNTHESYS program, projects FR-TAF 6972 and GB-TAF-6776, which supported this research during visits to Natural History Museum in London and Muséum national d'Histoire naturelle in Paris in 2017 to study historical type material.
Please select the country of your main research funder (please select carefully as in some cases this is used in fee calculation).	GERMANY - DE

as follow-up to "**Financial Disclosure**<br><br>Enter a financial disclosure statement that describes the sources of funding for the work included in this submission. Review the submission guidelines for detailed requirements. View published research articles from *PLOS ONE* for specific examples.<br><br>This statement is required for submission and **will appear in the published article** if the submission is accepted. Please make sure it is accurate.<br><br>**Funded studies**<br>Enter a statement with the following details:<br>• Initials of the authors who received each award<br>• Grant numbers awarded to each author<br>• The full name of each funder<br>• URL of each funder website<br>• Did the sponsors or funders play any role in the study design, data collection and analysis, decision to publish, or preparation of the manuscript?<br><br>Did you receive funding for this work?"	
**Competing Interests**<br><br>Use the instructions below to enter a competing interest statement for this submission. On behalf of all authors, disclose any competing interests that could be perceived to bias this work—acknowledging all financial support and any other relevant financial or non-financial competing interests.<br><br>This statement is required for submission and **will appear in the published article** if the submission is accepted. Please make sure it is accurate and that any funding sources listed in your Funding Information later in the submission form are also declared in your Financial Disclosure statement.	The authors have declared that no competing interests exist.

View published research articles from *PLOS ONE* for specific examples.<br><br>**NO authors have competing interests**<br><br>Enter: *The authors have declared that no competing interests exist.*<br><br>**Authors with competing interests**<br><br>Enter competing interest details beginning with this statement:<br><br>*I have read the journal's policy and the authors of this manuscript have the following competing interests: [insert competing interests here]*<br><br>* typeset	
**Ethics Statement**<br><br>Enter an ethics statement for this submission. This statement is required if the study involved:<br><br>• Human participants<br>• Human specimens or tissue<br>• Vertebrate animals or cephalopods<br>• Vertebrate embryos or tissues<br>• Field research<br><br>Write "N/A" if the submission does not require an ethics statement.<br><br>General guidance is provided below. Consult the submission guidelines for detailed instructions. **Make sure that all information entered here is included in the Methods section of the manuscript.**	The material collected in Ecuador is supported by the permit “Diversity distribution and evolution of the beetles of Ecuador - multidisciplinary approach with emphasis on systematics genetics and biogeography” (MAATE-DBI-CM--2022-0255) issued by the Ministry of Environment, Water and Ecological Transition of Ecuador.

Powered by Editorial Manager® and ProduXion Manager® from Aries Systems Corporation

**Format for specific study types**<br><br>**Human Subject Research (involving human participants and/or tissue)**<br>• Give the name of the institutional review board or ethics committee that approved the study<br>• Include the approval number and/or a statement indicating approval of this research<br>• Indicate the form of consent obtained (written/oral) or the reason that consent was not obtained (e.g. the data were analyzed anonymously)<br><br>**Animal Research (involving vertebrate animals, embryos or tissues)**<br>• Provide the name of the Institutional Animal Care and Use Committee (IACUC) or other relevant ethics board that reviewed the study protocol, and indicate whether they approved this research or granted a formal waiver of ethical approval<br>• Include an approval number if one was obtained<br>• If the study involved *non-human primates*, add *additional details* about animal welfare and steps taken to ameliorate suffering<br>• If anesthesia, euthanasia, or any kind of animal sacrifice is part of the study, include briefly which substances and/or methods were applied<br><br>**Field Research**<br><br>Include the following details if this study involves the collection of plant, animal, or other materials from a natural setting:<br>• Field permit number<br>• Name of the institution or relevant body that granted permission	
**Data Availability**<br><br>Authors are required to make all data underlying the findings described fully available, without restriction, and from the time of publication. PLOS allows rare exceptions to address legal and ethical concerns. See the PLOS Data Policy and FAQ for detailed information.	Yes - all data are fully available without restriction

A Data Availability Statement describing where the data can be found is required at submission. Your answers to this question constitute the Data Availability Statement and **will be published in the article**, if accepted.<br><br>**Important:** Stating 'data available on request from the author' is not sufficient. If your data are only available upon request, select 'No' for the first question and explain your exceptional situation in the text box.<br><br>Do the authors confirm that all data underlying the findings described in their manuscript are fully available without restriction?	
**Describe where the data may be found in full sentences. If you are copying our sample text, replace any instances of XXX with the appropriate details.**<br><br>• If the data are **held or will be held in a public repository**, include URLs, accession numbers or DOIs. If this information will only be available after acceptance, indicate this by ticking the box below. For example: *All XXX files are available from the XXX database (accession number(s) XXX, XXX.).*<br>• If the data are all contained **within the manuscript and/or Supporting Information files**, enter the following: *All relevant data are within the manuscript and its Supporting Information files.*<br>• If neither of these applies but you are able to provide **details of access elsewhere**, with or without limitations, please do so. For example:<br><br>*Data cannot be shared publicly because of [XXX]. Data are available from the XXX Institutional Data Access / Ethics Committee (contact via XXX) for researchers who meet the criteria for access to confidential data.*<br><br>*The data underlying the results presented in the study are available from (include the name of the third party*	The data presented here have been deposited at Dryad (datadryad.org). The locality as well as sequence data are also available in the Barcode of Life Data System ("BOLD", https://www.boldsystems.org).

and contact information or URL).<br>• This text is appropriate if the data are owned by a third party and authors do not have permission to share the data.<br><br>* typeset	
Additional data availability information:	Tick here if the URLs/accession numbers/DOIs will be available only after acceptance of the manuscript for publication so that we can ensure their inclusion before publication.

Powered by Editorial Manager® and ProduXion Manager® from Aries Systems Corporation

Manuscript
Click here to access/download;Manuscript;Balke_Liodessus_20240321_sub

Recent range expansion and lineage idiosyncratic population structure of *Liodessus* diving beetles in the high Andes (Coleoptera: Dytiscidae, Bidessini)

urn:lsid:zoobank.org:pub:296109F0-6DEE-4480-9B4B-DAE67A047596

Michael Balke[1,2], Tobias Mainda[3], Katja Neven[1], Lars Hendrich[1], Michael Steven Basantes[4], Carlos Prieto[5,6] & Adrián Villastrigo[1*]

1: SNSB-Zoologische Staatssammlung München, Münchhausenstraße 21, D-81247 Munich, Germany

2: GeoBioCenter, Ludwig Maximilians University, Munich, Germany

3: Zoological Institute and Museum, University of Greifswald, Greifswald, Germany, tobias.mainda@gmx.de

4: Entomology Division, Instituto Nacional de Biodiversidad INABIO, Pasaje Rumipamba 341 y Avenida de los Shyris, 170505 Quito, Ecuador

5: Departamento de Biología, Universidad del Atlántico, Barranquilla, Colombia

6: Corporación Universitaria Autónoma del Cauca, Popayán, Colombia

*Corresponding author: adrianvillastrigo@gmail.com

Abstract

Here, we review the taxonomy and population structure of diving beetles in the genus *Liodessus* Guignot, 1939 from the high Andes of southern Colombia and Ecuador. *Liodessus quillacinga ecuadoriensis* ssp. nov. is described from several localities in Ecuador (Type locality: Otavalo, Laguna San Pablo). *Liodessus quimbaya azufralis* Megna, Hendrich & Balke, 2019 stat. nov. is now regarded as a subspecies of *Liodessus quimbaya* Megna, Hendrich & Balke, 2019 based on new morphological and genetical data. *Liodessus quimbaya paletara* ssp. nov. is described from the Paletará Valley (Colombia: Cauca).

Key words

Colombia, Ecuador, Dytiscidae, Andes, *Liodessus*, new subspecies

Introduction

The Northern Andes have a diverse topography, characterized by strong climatic disparities across a relatively small region [1,2]. The vast, isolated high-altitude area of the Northern Andes, with its large intramontane depressions, is covered by the unique tundra-alpine Páramo ecosystem between latitudes 11°N and 8°S. Páramos extend over an area of 35,000 km^2 at altitudes between 2,800 and ca. 4,700 m [3]. Already marvelled by von Humboldt [4], these regions are now acknowledged as botanical biodiversity hotspots, hosting around 5,000 almost exclusive endemic plant species [5]. Over the past million years, the Colombian Páramos have been subjected to a dynamic series of connection and disconnection events driven by alternating glacial and interglacial periods, which is referred to as a "flickering connectivity system" [2]. This intricate process has not only impacted the interconnectivity of major mountain ranges, but has also influenced the connectivity of Páramo areas. Consequently, it has given rise to complex speciation processes shaped by the dynamic interactions of populations and their

dispersal capabilities. Furthermore, the reduction in wing size, a common insect adaptation to high elevation [6], may curtail dispersal capabilities. Recent studies indicate a complex interplay of biotic and extrinsic factors, along with diversity-dependent processes, which could determine the shaping of diversity patterns and the distribution of mountain species, e.g. [7-9]. Notably, recent attention has been devoted to high Andean diving beetle communities, particularly those belonging to the genus *Liodessus*, Guignot, 1939, e.g. [10].

Liodessus, a genus of the tribe Bidessini (Dytiscidae, Hydroporinae), contains beetles characterized by their diminutive size – predominantly smaller than 3 mm, and their distribution in the Neotropical and Nearctic regions [11]. It is noteworthy that Afrotropical species currently assigned to this genus [12] represent in fact a different, not closely related lineage (Balke et al., unpublished). Within the Andes, *Liodessus* are found from lowlands to nearly 5,000 m, primarily inhabiting lotic habitats (rarely slow-flowing channels) where they often constitute the most abundant aquatic beetles [13, 14]. Despite their prevalence, *Liodessus* from the high-altitude Páramo and Puna ecoregions remained poorly studied. Over the past few years, numerous reports on *Liodessus* from these regions have been published, including descriptions of new taxa and documentation of new records, e.g. [10, 13-16]. To support and enhance morphological results, and better understand distribution patterns and phylogenetic affinities of *Liodessus* taxa, the public COLLI project ("Colombian and Andean diving beetles in the genus *Liodessus*", see [17] for details) was initiated, based on the Barcode of Life Data System (BOLD) of the Canadian Centre for DNA Barcoding, specifically targeting the 5' end of the mitochondrial cytochrome *c* oxidase subunit I (5-COI) gene fragment [18]. COLLI currently contains 466 sequences for the 5-COI for more than 30 taxa, and these numbers are constantly growing due to the very active nature of this database, with ongoing additions of sequence data and taxonomic curation.

To date, only *Liodessus riveti* Peschet, 1923 [19] was reported from Ecuador (see below). On the other hand, more attention has been focused on the fauna of the Colombian Andes, with six species plus seven subspecies currently recognized 10]. The same is true for the Andean fauna of Peru, south of Ecuador, with seven described species [11].

Balke et al. [10] introduced an updated interpretation of the *Liodessus* fauna of the eastern branch of the Colombian Andes, suggesting the *L. bogotensis*-complex, for populations widespread along this section of the Andes. This complex now contains various subspecies introduced to account for genetic structure and in some cases subtle morphological differentiation related to different Páramo or Altiplano areas (geographic structure). In alignment with this methodology, we combined morphological analyses with 5-COI sequencing to present the first comprehensive assessment of the high Andean aquatic beetle fauna in northern Ecuador and provide a more comprehensive assessment of the (southern) Colombian fauna. The specific objectives are (1) taxonomic identification, (2) and re-assessment of described taxa, (3) providing a refined geographic interpretation and (4) the revealing of the population genetic structure within the studied taxa.

Material and methods

Sampling and map generation

New samples were acquired in the framework of a large-scale Andean water beetle research project (headed by Michael Balke in cooperation with researchers from across south America). In Ecuador, new collections were obtained by Michael Basantes (INABIO, Quito) and Michael Balke; in Colombia by Carlos Prieto (Universidad del Atlántico, Barranquilla). A map was generated using QGIS v3.16.4 (www.qgis.org) using the Natural Earth raster map as base layer.

Locality information and species lineage (sensu molecular information, see below) was represented by using a categorized visualization proportional to the sample size.

Acronyms

INABIO	Instituto Nacional de Biodiversidad, Quito, Ecuador,
LIAUN	Laboratorio de Insectos Acuáticos, Departamento de Biología, Universidad Nacional de Colombia,
UNAL	Colombian National Collecion of Entomology, ICN, Universidad Nacional de Colombia,
ZSM	SNSB-Zoologische Staatssammlung, Munich, Germany, vouchers temporarily stored for further morphological work.

Codes in the format ECU_MB_2022_01 in the studied material sections are ZSM locality codes, and refer to the country of origin (ECU = Ecuador; COL = Colombia), collector who organized the fieldwork (cPr = Carlos Prieto, MB = Michael Balke, MSB = Michael Steven Basantes, YSM = Yoandri Suarez Megna), year of collection (e.g., 2022) and a locality number for the respective collecting event (e.g., 01).

Morphological descriptions and photography

The minimalistic description format of morphological characters follows previous publications on *Liodessus*, e.g. [14]. Images were taken using a Canon EOS R camera. A Mitutoyo 10x ELWD Plan Apo objective was used for the habitus, and a corresponding 20x objective for the genital structures. These objectives were attached to a Carl Zeiss Jena Sonnar 3.5/135 MC as focus lens. Three SN-1 LED segments from Stonemaster were used for illumination (www.stonemaster-onlineshop.de). Image stacks were created using a Stackmaster macro rail

(Stonemaster). The stacks were fused using Helicon Focus v. 7.61 on a MacPro 2019 (Apple Inc.) with a Radeon Pro 6800X MPX GPU.

DNA sequencing

The laboratory protocol for DNA sequencing and subsequent data analysis follows the Canadian Centre for DNA Barcoding (CCDB) standard procedures for barcoding (ww.ccdb.ca). Tissue samples were processed at CCDB, with the resulting barcode sequences (COI-5) being uploaded to the BOLD-system. To assess the relationship between newly acquired sequences and existing species, a straightforward approach was followed: a neighbour-joining tree, based on *p*-distances, was computed using Geneious v11.0.4 [20]. This methodology has been used in recent studies on *Liodessus* (e.g. [10]) to support the description process based on morphological data (in combination with molecular and geographical evidence).

Trees, haplotype networks and the calculation of genetic distance

Nucleotide sequences of 206 specimens, belonging to the taxa referred above, were retrieved from BOLD-system and subjected to alignment using MAFFT v7.450 [21] with the AUTO algorithm, as implemented in Geneious v11.04 software. For quality control, the nucleotide alignments were translated into amino acid sequences, allowing for visual inspection to identify potential sequencing errors, specifically internal stop codons. This step served as a crucial measure to ensure the accuracy and reliability of the data. To better comprehend the information content embedded in the nucleotide data, the Geneious tree builder was employed by using the neighbour joining method with the Jukes Cantor genetic distances. Nucleotide DNA alignments were used to estimate haplotypes using PHASE [22] through 1,000 iterations. A burn-in fraction of 10% and a thinning parameter of 5 were applied during this process to ensure robust results.

Subsequently, haplotype networks were constructed using TCS software [23], considering a connection threshold of 95% to generate a fully connected network. For enhanced visualization of the haplotype network, tcsBU [24] was used considering collecting sites used as groups. Pairwise genetic differentiation among population was explored in DNAsp v6.12.03 [25]. The fixation index (Fst) was calculated as a metric for assessing connectivity between different populations. Visualization of the Fst values was represented as a heatmap using Python 3 [26] complemented by the Seaborn library [27].

Diagnostic characters

The aligned nucleotide sequences were analysed for each species in Geneious v11.04 using the "Highlighting > All disagreement to consensus" function. The diagnostic characters were visually inspected to identify unique consensus bases whose position in the multiple sequence alignment for each of the sublineages.

Results

Taxonomy

Liodessus quillacinga quillacinga Megna, Hendrich & Balke, 2019

Figs 1, 2, 3A, 4A, 5B, 6, 7

Type locality. Colombia: Nariño, Pasto, surroundings of the Galeras volcano, 1.166° -77.333°.

Holotype. Colombia: Nariño, Pasto, surroundings of the Galeras volcano, 3,200 m, 05.v.2017, 1.166° -77.333°, Megna & Ruíz (CO-20) (LIAUN). **Paratypes.** See Megna et al. [16].

Description of holotype. Total length 2.0 mm; length without head 1.7 mm; maximum width 0.9 mm. Shape elongate, oval with lateral outline discontinuous between pronotum and elytron. Pronotum broadest in its midlength, sites convex. Elytra widest at about midlength.

Colour. Dorsally and ventrally testaceous to dark reddish-brown. Antenna, mouthparts, pro-, meso- and metathoracic legs testaceous (Fig 3A).

Surface sculpture. Head with distinct microreticulation and few punctures. Pronotum shiny, anteriorly with faint microreticulation; with dense unevenly distributed coarse setiferous punctation. Elytron shiny; surface with dense and coarse setiferous punctation.

Structures. Head with distinct occipital line. Pronotum with lateral bead; with distinct and very deep basal striae. Elytron with short and deep basal striae.

Genitalia. Median lobe of aedeagus in lateral view simply curved, broadened medially, in ventral view lateral margins parallel basally and strongly narrowed.

Metathoracic wings. Vestigial, reduced to short membranous stubs without sign of venation.

Variation. (N = 11) Total length 2.0-2.2 mm; length without head 1.7–1.9 mm; maximum width 0.9–1.0 mm. The extent of the basal elytral striae varies from slightly to well expressed. The dorsal coloration can be slightly darker, laterally on pronotum and elytron in some specimens. The structure of median lobe of the aedeagus in lateral and ventral view is constant in general shape as illustrated in Figs 4A and 5B.

Female. Dorsal side dull, with strong microreticulation.

Identification notes. In the COLLI sequence database, *L. quillacinga quillacinga* has two diagnostic characters different from the other sublineages of *L. quillacinga* (Table 1). The haplotype network structure is depicted in Fig 6.

Distribution. To date only known from its type locality in the Pasto Massif in southern Colombia at an altitude of 3,200 m (Fig 2A, B).

Habitat. Unshaded permanent lagoon with aquatic vegetation (Fig 10b Megna et al. [16]).

Table 1. Nucleotide diagnostic characters in the COI alignment for sublineages of *Liodessus quillacinga* (above) and *Liodessus quimbaya* (below).

**Taxon, Number of sequences**	**28**	**88**	**202**	**316**	**322**	**325**	**343**	**433**	**628**					
L. quillacinga quillacinga (5)		C		G										
L. quillacinga cumbalis (5)		C	C	G				G						
L. quillacinga cochaensis (5)						A	T							
L. quillacinga ecuadoriensis (44)	G				G				A/G					
**Taxon, Number of sequences**	**40**	**55**	**124**	**178**	**229**	**259**	**271**	**283**	**298**	**325**	**440**	**472**	**494**	**592**
L. quimbaya quimbaya (24)	G	A												
L. quimbaya quimbaya (106)			G		C	G	A		C	A(G)	T		T	
L. quimbaya paletara (20)				G				G				G		G(A)

Liodessus quillacinga cochaensis Megna, Hendrich & Balke, 2019

Figs 1, 2, 3B, 4B, 5C, 6, 7

Type locality. Colombia: Nariño, Pasto, Vereda El Motilón, Cocha lagoon, 1.116° -77.166°.

Holotype. Colombia: Nariño, Pasto, Vereda El Motilón, Cocha lagoon, 2,790 m, 02.v.2017, 1.166° -77.166°, Megna & Ruíz (CO-17) (LIAUN). **Paratypes.** See Megna et al. [16].

Size of holotype. Total length 1.9 mm; length without head 1.6 mm; maximum width 0.9 mm.

Variation. (N = 9) Total length 1.9–2.2 mm; length without head 1.6–1.9 mm; maximum width 0.9–1.0 mm.

Metathoracic wings. Fully developed.

Diagnosis. This subspecies is very similar to other populations, it may be separated from *L. quillacinga quillacinga* and from *L. quillacinga cumbalis* by having irregular maculations on the elytra (Fig 3B). From *L. quillacinga ecuadoriensis* ssp. nov., it may be separated by the

median lobe of aedeagus (Figs 4B, 5C). This subspecies is characterized geographically, known only from its type locality in southern Colombia. In the COLLI sequence database, *L. quillacinga cochaensis* has two diagnostic characters different from the other sublineages (Table 1). The haplotype network structure is depicted in Fig 6.

Distribution. To date only known from its type locality in the Pasto Massif of southern Colombia at an altitude of 2,790 m (Fig 2A, B).

Habitat. Permanent, exposed lagoon with aquatic plants.

Liodessus quillacinga cumbalis Megna, Hendrich & Balke, 2019

Figs 1, 2, 3C, 4C, 5A, 6, 7

Type locality. Colombia: Nariño, Túquerres, surroundings of the Cumbal volcano, Vereda Qulismar, 1.091° -77.718°. **Holotype.** Colombia: Nariño, Túquerres, surroundings of the Cumbal volcano, Vereda Qulismar, 3,840 m, 10.v.2017, 1.091° -77.718°, Megna & Ruíz (CO-28) (LIAUN). **Paratypes.** See Megna et al. [16].

Size of holotype. Total length 2.0 mm; length without head 1.7 mm; maximum width 0.9 mm.

Variation. ($N = 4$) Total length 2.0–2.2 mm; length without head 1.7–1.9 mm; maximum width 0.9–1.0 mm.

Metathoracic wings. Fully developed.

Diagnosis. This subspecies is very similar to other populations, it may be separated from *L. quillacinga quillacinga* and from *L. quillacinga cumbalis* by having generally darker elytra with less maculations (Fig 3C). From *L. quillacinga ecuadoriensis* ssp. nov., it may be separated by the median lobe of aedeagus (Figs 4C, 5A). This subspecies is characterized geographically, known only from its type locality in southern Colombia. In the COLLI sequence database, *L. quillacinga cumbalis* has four diagnostic characters different from the other sublineages (Table 1). The haplotype network structure is depicted in Fig 6.

Distribution. To date only known from the surroundings of the Cumbal volcano in the Pasto Massif of southern Colombia at an altitude of 3,840 m (Fig 2A, B).

Habitat. Páramo bog with muddy soil and submerged vegetation.

Liodessus quillacinga ecuadoriensis ssp. nov.

Figs 1, 2, 3D–F, 4D–G, 5D–H, 6, 7, 8A–D, 9, 10A–C

urn:lsid:zoobank.org:act:E86478D4-F4F9-4311-A59F-FAB6DA66BFE3

Type locality. Ecuador: Imbabura, Otavalo, Laguna San Pablo, 0.199° -78.2331°. **Holotype.** Ecuador: Imbabura, Otavalo, Laguna San Pablo, humedal, 2,700 m, 28.xi.2022, 0.199° -78.2331°, Basantes & Balke (ECU_MB_2022_10) (INABIO). **Paratypes.** 334 specimens with same data as the holotype (INABIO, ZSM); 75 exs., Ecuador: Pichincha, Cotopaxi, small wet patch 3,700 m, 21.xi.2022, -0.5657° -78.4438°, Basantes & Balke (ECU_MB_2022_01) (ZSM); 246 exs., Ecuador: Napo, Antisana, La Mica area, 3,950 m, 22.xi.2022, -0.5361° 78.231°, Basantes & Balke (ECU_MB_2022_02) (INABIO, ZSM); 165 exs., Ecuador: Imbabura, Laguna Mojanda, wet plant carpet, 3,750 m, 27.xi.2022, 0.1338° -78.2598°, Basantes & Balke (ECU_MB_2022_09) (INABIO, ZSM).

Size of holotype. Total length 2.0 mm; length without head 1.8 mm; maximum width 1.0 mm.

Variation. (*N* = 41). Total length 1.8–2.3 mm; length without head 1.5–2.0 mm; maximum width 0.9–1.1 mm.

Metathoracic wings. Fully developed.

Male genitalia. Median lobe of aedeagus ventrally slim. In lateral view, simply curved parallel-sided, without broadened medially. In ventral view, lateral margins parallel basally, and in the median third conically narrowed towards the tip (Figs 4D–G, 5D–H).

Diagnosis. This new subspecies is very similar to other populations and can only be reliably separated on the basis of the locality and 5-COI data. Its aedeagus is ventrally and laterally slimmer and more parallel-sided. Ventrally, the median third is more distinct conically

narrowed towards the tip. Laterally, it is not medially broadened than in *L. quillacinga quallacinga* and its two other subspecies (Figs 4 and 5). The coloration of the elytra shows a high variability (Figs 3D–F, 8A–D and 9). *Liodessus quillacinga ecuadoriensis* ssp. nov. is characterized geographically, known from several localities in northern Ecuador. In the COLLI sequence database, this new subspecies has three diagnostic characters different from the other sublineages (Table 1). The haplotype network structure is depicted in Fig. 6

Distribution. *Liodessus quillacinga ecuadoriensis* ssp. nov. is distributed at altitudes between 2,700 and 3,950 m in northern Ecuador (Fig 2).

Habitat. Collected from very shallow water with dense vegetation, even small saucer sized water accumulations around lagoons (Fig 10A–C).

Etymology. With the choice of the epithet "*ecuadoriensis*", the new subspecies is named after the country of Ecuador, which includes the type localities. The name is an adjective in the nominative singular.

Liodessus quimbaya quimbaya Megna, Hendrich & Balke, 2019

Figs 11, 12, 13A, 14E, 15B, 16A, D, 17, 19

Type locality. Colombia: Quindío, Salento, Paramo Frontino, 4.608° -75.428°. **Holotype.** Colombia, Quindío, Salento, Paramo Frontino, 3,700 m, 25.iv.2017, 4.608° -75.428°, Megna, Osorio & Vivero (CO-14) (LIAUN). **Paratypes.** See Megna et al. [16].

Additional material. 23 exs., Colombia, Cauca, Malvaza, 3,500 m, 29.i.2019, 2.483° -76.233°, Prieto (COL_CPr_2019_01) (UNAL, ZSM).

Description of holotype. Total length 2.0 mm; length without head 1.8 mm; maximum width 1.0 mm. Shape elongate, with lateral outline only slightly discontinuous between pronotum and elytron. Pronotum broadest in its midlength, sides not very convex. Elytra widest at about midlength.

Colour. Dorsal side and ventrally dark brown to black. Antenna, mouthparts, pro-, and mesothoracic legs testaceous. Metathoracic leg and antennomeres largely infuscate.

Surface sculpture. Head with distinct microreticulation and few punctures. Pronotum mostly shiny, anteriorly with faint microreticulation; with unevenly distributed coarse setiferous punctation. Elytron shiny; surface with dense and coarse setiferous punctation.

Structures. Head with distinct occipital line. Pronotum with lateral bead; with distinct and very deep basal striae. Elytron with short and hardly defined basal striae.

Genitalia. Median lobe of aedeagus in lateral view longish, simply curved, apically narrowed, in ventral view lateral margins slightly narrowed medially and tip narrowed.

Metathoracic wings. Vestigial, reduced to short membranous stubs without sign of venation.

Variation. Total length 1.9–2.2 mm (*N* = 22); length without head 1.6–1.9 mm; maximum width 0.9–1.0 mm. The extent of the basal elytral striae varies from absent to fairly well expressed. The dorsal coloration can be brighter, dark orange, laterally on pronotum and elytron in some specimens. The structure of median lobe of the aedeagus in lateral and ventral view is constant in general shape but the curvature varies as illustrated in Figs 14E, 15B, 16A, D.

Female. Dorsal side dull, with strong microreticulation.

Identification notes. In the COLLI sequence database, *L. quimbaya quimbaya* has 2 diagnostic characters different from the other sublineages (Table 1). The haplotype network structure is depicted in Fig 18.

Distribution. To date only known from the Central Cordillera in southern Colombia at altitudes between 3,290 and 3,700 m (Fig 11).

Habitat. Páramo water body with compact aquatic vegetation, Fig 11a in Megna et al. [16].

Liodessus quimbaya azufralis Megna, Hendrich & Balke, 2019 stat. nov.

Figs 11, 12, 13A, C–D, 14C–D, 15A–D, 17B, E–I, 18, 19

Type locality. Colombia, Narino, Surroundings of the Azufral volcano, 1.087°, -77.711°. **Holotype.** Colombia, Narino, Municipality of Túquerres, Surroundings of the Azufral volcano, 3,795 m, 07.v.2017, 1.087°, -77.711°, Megna & Ruiz (CO-25) (LIAUN). **Paratypes.** See Megna et al. [16].

Additional material. 197 exs., Ecuador: Napo, Coyambe Coca, puddle, 3,700 m, 23.xi.2022, -0.3169° -78.1423°, Basantes & Balke (ECU_MB_2022_03) (INABIO); 182 exs., "Ecuador: Napo, Coyambe Coca, small round lagoon, 3,900 m, 24.xi.2022, -0.3223° -78.1583°, Basantes & Balke (ECU_MB_2022_04) (INABIO, ZSM); 141 exs., "Ecuador: Napo, PN Coyambe Coca, small lagoon, 4,000 m, 26.xi.2022, -0.3007° -78.1338°, Basantes & Balke (ECU_MB_2022_06) (INABIO, ZSM); 111 exs., Ecuador: Napo, PN Coyambe Coca, puddle, 3,950 m, 26.xi.2022, -0.3018° -78.1303°, Basantes & Balke (ECU_MB_2022_07) (INABIO, ZSM); 210 exs., Ecuador: Carchi, Ecological Reserve El Ángel, 3,750 m, 14.ii.2023, 0.6854° -77.8766°, Basantes (ECU_MSB_2023_01) (INABIO, ZSM); 329 exs., Ecuador: Carchi, Ecological Reserve El Ángel (Lagunas verdes), 3,950 m, 15.ii.2023, 0.7991° -77.9279°, Basantes (ECU_MSB_2023_03) (INABIO, ZSM); 108 exs., Ecuador: Carchi, Biological Station Guandera (nearby puddles), 2,750 m, 16.ii.2023, 0.59522° -77.78842°, Basantes (ECU_MSB_2023_04) (INABIO, ZSM).

Note. Megna, Hendrich & Balke (in Megna et al. [16]) described *L. quimbaya* and *L. azufralis* at species level. In particular, morphological analysis of new material from further localities indicates a much greater similarity of the two taxa than assumed in the original description. Therefore, it is suggested that both taxa belong to one species, with one subspecies and names made available in the same paper (Art. 8, Art. 10., Art. 11, Art. 16, Art. 23, ICZN [28]). As *L. quimbaya* was described on pages 10ff. and *L. azufralis* on pages 14ff. of Megna et al. [16], *L. quimbaya* is chosen as priority name for this taxon, acting as "First Revising Author" according to Art. 24.2 of ICZN [28]. Accordingly, the new combination must be *L. quimbaya azufralis* stat. nov. for this taxon.

Size of holotype. Total length 2.1 mm; length without head 1.8 mm; maximum width 1.0 mm.

Variation. Total length 1.7–2.1 mm (*N* = 60); length without head 1.5–1.9 mm; maximum width 0.8–1.0 mm.

Metathoracic wings. Vestigial, reduced to short membranous stubs without sign of venation in random specimens dissected from all populations.

Diagnosis. This subspecies is morphologically very similar to other populations. The habitus of *L. quimbaya azufralis* stat. nov. is however more broadly oval and drop shaped than in *L. quimbaya quimbaya* and *L. quimbaya paletara* ssp. nov. (Figs 13, 14). The dorsal coloration is mostly brighter. This subspecies is characterized geographically, occurring in southern Colombia as well as northern Ecuador. In the COLLI sequence database, this subspecies has eight diagnostic characters different from the other sublineages (Table 1). The haplotype network structure is depicted in Fig 18.

Distribution. *Liodessus quimbaya azufralis* stat. nov. is widely distributed in the Pasto Massif of southern Colombia and northern Ecuador at altitudes between 2,700 and 4,000 m (Fig 12).

Habitat. Mostly collected from very shallow water with dense vegetation, even small saucer sized water accumulations around lagoons or in small depressions along the road; also collected from deeper water (up to 40 cm) at along the edge of a small, round lagoon, with green algae and vegetation at the edge and body of the lagoon.

Liodessus quimbaya paletara ssp. nov.

Figs 11, 12, 14B, 15F, 16A, 17C, 18, 19

urn:lsid:zoobank.org:act:D9FB958C-1F95-4D23-ACC3-0F5BDB1439AD

Type locality. Colombia, Cauca, Paletará Valley, 2.167° -76.466°. **Holotype.** Colombia, Cauca, Paletará, 2,850 m, 30.iv.2019, 2.167° -76.466°, Prieto (COL_CPr_2019_03) (UNAL, ZSM). **Paratypes.** 134 exs., same label data (UNAL, ZSM); 82 exs., Colombia, Cauca, Paletará, 2,900 m, 11.ix.2019, 2.167° -76.466°, Prieto (COL_CPr_2019_02) (UNAL, ZSM).

Size of holotype. Total length 1.9 mm; length without head 1.6 mm; maximum width 0.9 mm.

Variation. (*N* = 11) Total length 1.9–2.0 mm; length without head 1.6–1.7 mm; maximum width 0.9 mm.

Metathoracic wings. Vestigial, reduced to short membranous stubs without sign of venation in random dissected specimens from both localities.

Diagnosis. This new subspecies is very similar to other populations. It is well-characterized geographically. In the COLLI sequence database, this new subspecies has four diagnostic characters different from the other sublineages (Table 1). The haplotype network structure is depicted in Fig 18.

Distribution. To date only known from the Paletará Valley in the Central Cordillera of southern Colombia at altitudes between 2,850 and 2,900 m (Fig 12).

Habitat. Collected from very shallow water with dense vegetation next to the road from Paletará to San José de Isnos.

Etymology. With the choice of the epithet "*paletara*", this new subspecies is named after its type locality, the Paletará Valley in the Colombian Massif. The name is a noun in the nominative singular standing in apposition.

Notes on *Liodessus riveti* Peschet, 1923

Liodessus riveti Peschet, 1923 was described from Ecuador, data: El Pelado, 4,150 m, January 1903, leg. P. Rivet. The holotype is deposited in the Museum National d'Histoire Naturelle in Paris [19], but could not be located during a stay of M. Balke in 2017. Without re-examination and only based on the original description, no reliable taxonomic assessment can be made.

In the World Catalogue of Dytiscidae [11], the type locality is given as El Pelado (island), referring to an islet in the El Pelado marine reserve of Ecuador. This is most likely incorrect, as the altitude given by Peschet[19] indicates an alpine area. Paul Rivet did in fact explore the high-

altitude regions of Ecuador, and in January 1903, he worked in the area of Tulcán in northern Ecuador [29]. It seems adequate to infer that El Pelado refers to the Cerro El Pelado, part of the Reserva Ecológica El Ángel, situated at 0.7268° -77.9219°.

The team of Michael Balke has made extensive collections in that reserve recently. The *Liodessus* collected there were all assigned to *L. quimbaya azufralis* stat. nov. (see above), so that re-examination of the type of *L. riveti* might reveal the conspecificity of both taxa. This would make *L. quimbaya* a younger synonym of *L. riveti* and lead to the corresponding renaming of its herein discussed sublineages (Art. 23, ICZN [28]).

Molecular evidence

The complete DNA alignments comprise DNA sequences of 147 specimens of *Liodessus quimbaya* and 59 specimens of *Liodessus quillacinga*, for which 124 and 44 are newly sequenced respectively. Neighbour joining trees (Figs 1, 11) and haplotype networks (Figs 6, 19) provide complementary and compatible data, clustering sequences/haplotypes in various molecular operational taxonomical units that we have considered as valid subspecies (in agreement with the morphological and geographical evidence).

Liodessus quillacinga

The neighbour-joining tree (Fig 1) breaks down the samples into four distinct groups, in agreement with the four geographically structured clusters identified in the haplotype network (Fig 6). The three *L. quillacinga* subspecies, as defined by Megna et al. [16], are each represented by isolated localities in southern Colombia, whereas the newly described *L. quillacinga ecuadoriensis* ssp. nov. corresponds to a wider geographic sampling from northern Ecuador. Pairwise comparison of *L. quillacinga* localities (Fig 7) underscores substantial isolation and differentiation for each subspecies (Fst ≥ 0.83). In contrast, *L. quillacinga*

ecuadoriensis ssp. nov. exhibits robust intrapopulation connectivity (Fst < 0.73), with the majority of comparisons below 0.51, indicating a comparatively lower level of differentiation and a higher level of gene flow between populations.

Liodessus quimbaya

In the case of *L. quimbaya*, the neighbour-joining tree shows a distinct clustering of localities into three geographically structured groups (Fig 11). Specifically, samples from Ecuador and the Colombian locality near the Ecuadorian border (Túquerres) group together. This group (*L. quimbaya azufralis* stat. nov.) shows a robust intrapopulation connectivity (Fst ≤ 0.84). Samples collected from Paletará (Colombia) constitute a separate clade (*L. quimbaya paletara* ssp. nov.). The remaining samples, situated in the northern region of the sampling area, form the third distinct group. As can be seen in the haplotype network (Fig 18), these three clusters match the three subspecies identified in this study. The connectivity pattern exhibited by the heatmap (Fig 19) is not as clear as is in *L. quillacinga*, yet it reveals some level of isolation and differentiation at both intra- and interpopulation levels. Nonetheless, it highlights a comparatively lower genetic differentiation (indicated as colder colours in the heatmap) within each subspecies.

Updated checklist of *Liodessus* from Colombia and Ecuador

Liodessus bogotensis bogotensis Guignot, 1953 (Colombia)

Liodessus b. almorzadero Balke et al., 2023 (Colombia)

Liodessus b. chingaza Balke et al., 2023 (Colombia)

Liodessus b. lacuniviridis Balke et al., 2020 (Colombia)

Liodessus b. matarredonda Balke et al., 2023 (Colombia)

Liodessus b. sumapaz Balke et al., 2023 (Colombia)

Liodessus picinus Balke et al., 2021 (Colombia)

Liodessus quillacinga quillacinga Megna, Hendrich & Balke, 2019 (Colombia)

Liodessus quillacinga cochaensis Megna, Hendrich & Balke, 2019 (Colombia)

Liodessus quillacinga cumbalis Megna, Hendrich & Balke, 2019 (Colombia)

Liodessus quillacinga ecuadoriensis ssp. nov. (Ecuador)

Liodessus quimbaya quimbaya Megna, Hendrich & Balke, 2019 (Colombia)

Liodessus quimbaya azufralis Megna, Hendrich & Balke, 2019 stat. nov. (Colombia, Ecuador)

Liodessus quimbaya paletara ssp. nov. (Colombia)

Liodessus riveti Peschet, 1923 (Ecuador)

Liodessus santarosita Balke et al., 2023 (Colombia)

Discussion

The integrative approach employed here has identified taxonomically informative characteristics, at the same time emphasizing those susceptible to pronounced variability. Notably, we here find a greater intraspecific variability in the studied taxa than previously reported by Megna et al. [16], particularly concerning coloration and the extent of basal striae on pronotum and elytra. Molecular phylogenetic evidence revealed morphologically cryptic, geographically structured population-level diversity. The observed patterns find a potential explanation in the "flickering connectivity system" model of the Páramo ecosystem, outlined by Flantua et al. [2]. Shaped by the dynamic shifts between glacial and interglacial Pleistocene periods, the elevations at which Páramos developed underwent continuous changes over the last million years. This dynamism has fostered connections and disconnections among Páramos, influencing dispersal, gene flow and local extinction of Andean *Liodessus* species within Páramo ecosystems. This phenomenon may also account for recent speciation processes detected in the area, as well as the observed intraspecific variability and hybridization within the *Liodessus bogotensis* species complex – a related species from the eastern Cordillera of Colombia [10]. Notably, the "flickering connectivity system" plays a crucial role in shaping biodiversity across diverse taxa. This is underscored by its substantial influence on the present

diversity of plants in the Páramos (ca. 5,000 species [5]) or by the recent diversification of the majority of bird species in the Páramo [30], which emerged after the late Miocene/early Pliocene. In addition, differences in β-diversity were found to divide the Páramos of the northern Andes (Colombia) and the southern Andes (Ecuador) into two spatially structurally distinct groups, which is explained by past climatic conditions.

A fascinating pattern of disconnection emerges from the detailed study of what we classify as *Liodesus quimbaya* subspecies: while each subspecies exhibits moderate intralineage variability, their interlineage Fst values indicate a significant disconnection. Particularly intriguing is the proximity of *L. quimbaya quimbaya*, covering a known distribution range of over 250 km from north to south, to *L. quimbaya paletara* ssp. nov., separated by a mere 40 km at the southern limit. The presence of the active Puracé volcano in the distribution boundary of both subspecies may have intensified their isolation. The volcanic activity, as proposed by Finn et al. [31], emerges as a plausible environmental influence contributing to the observed genetic structure, likely wiping out some populations and generating local bottlenecks (i.e., reduction of the genetic pool) that leaved a lasting genetic imprint. However, this area presents a valuable opportunity for further research to evaluate the stability of the proposed *Liodessus* classification and explore the intraspecific boundaries of *Liodessus quimbaya*.

The present study significantly contributed to our understanding of the geographical range of *L. quimbaya azufralis* stat. nov. by expanding its known distribution to northern Ecuador. In contrast to *Liodessus quillacinga*, all subspecies of *Liodessus quimbaya* exhibit a notable reduction in wing size. Surprisingly, despite the anticipated impact of varying wing development on the potential dispersal capabilities (vestigial wings for *L. quimbaya* and fully developed wings for *L. quillacinga*), the latter (fully winged one) displays a higher number of lineages in a smaller geographical span. Interestingly, the findings indicate that the distinct

dispersal capabilities of the two species do not seem to be the primary factor influencing their diversity. While distance and dispersal capabilities are not the main factors, the overall higher connectivity of the Ecuadorian Páramos (sensu Flantua et al. [2]) supports the idea that the changing extend of Páramo over time helps to explain the genetic and slight morphological variations observed. In the case of *Liodessus quimbaya quimbaya*, there appears to be evident connectivity within the Colombia Central Cordillera between the Páramo districts of Viejo Caldas-Tolima and Macizo Colombiano allowing recent range expansion.

The flickering system, although not rigid, displays varying degrees of stability along the Andes, with the southern Andes (Ecuador) showing a higher degree of connectivity compared to the northern Andes in Colombia [2], occasionally interrupted by rare instances of fragmentation. This is supported by our results, suggesting the flickering connectivity system is a strong force driving intralineage connectivity and interlineage isolation, consequently fostering recent evolutionary expansion. The star-shaped [31-33] pattern of the haplotype clusters in the southern part of the geographic ranges in the sublineages of *L. quillacinga* and *L. quimbaya* indicates recent range expansion into the northern Ecuador region. Processes caused by the flickering system or genetic differentiation over time due to large geographical distances, could (re)separate populations in the future, hindering dispersal and gene flow. This, in turn, might lead to the evolution of new endemic species, potentially contributing to the development of cryptic diversity that have not yet have developed significant morphological differences [34, 35]. However, recent studies indicate that the evolution of different lineages does not necessarily have to be accompanied by the evolution of significant morphological differences, especially for species inhabiting extreme habitats, such as high alpine areas [36, 37]. Therefore, it is possible that the lineages discussed here are in the process of speciation, as illustrated by the high number of haplotypes for *L. quillacinga ecuadoriensis* ssp. nov. and *L. quimbaya azufralis* stat. nov. The observed patterns can be interpreted as "cryptic diversity", but whether this diversity will persist in the ongoing evolutionary process remains unclear. The isolated

lineages in Colombia, displaying cryptic diversity, do not currently indicate the evolution of significant morphological characters. It is possible that isolation and speciation are not yet complete and may never be, as long as anthropogenic climate change does not result in the absence of further dynamic changes between glacial and interglacial periods [38].

The recent range expansion and lineage idiosyncratic population structure of *Liodessus* diving beetles in the high Andes of Ecuador and Colombia seems to be closely linked to climatic processes. Further investigations, in particular with much finer tuned population genomic methods, could therefore not only lead to new taxonomic results, but also improve the understanding of the relationship between climate protection and species conservation - one of the greatest challenges of the 21st century.

References

1. Flantua SGA, Hooghiemstra H, Vuille M, Behling H, Carson JF, Gosling WD, et al. Climate variability and human impact in South America during the last 2000 years: synthesis and perspectives from pollen records. Climate of the Past 2016; 12: 483-523. doi: 10.5194/cp-12-483-2016.
2. Flantua SGA, O'Dea A, Onstein RE, Giraldo C, Hooghiemstra H. The flickering connectivity system of the north Andean páramos. Journal of Biogeography 2019; 46: 1808-1825. doi: 10.1111/jbi.13607.
3. Madriñán S, Cortés AJ, Richardson JE. Páramo is the world's fastest evolving and coolest biodiversity hotspot. Front. Genet. 2013; 4: 192. doi: 10.3389/fgene.2013.00192.
4. Humboldt A von. Reise in die Aequinoctial-Gegenden des neuen Continents in den Jahren 1799, 1800, 1801, 1803 und 1804, Erster Theil. Stuttgart, Tübingen, Cotta; 1815.

5. Peyre G, Balslev H, Font X. Phytoregionalisation of the Andean páramo. PeerJ. 2018; 6: e4786. doi: 10.7717/peerj.4786.
6. Mani MS. Ecology and Biogeography of High Altitude Insects. Dr. W. Junk N.V. Publishers, The Hague; 1968.
7. Condamine FL. Limited by the roof of the world: mountain radiations of Apollo swallowtails controlled by diversity-dependence processes. Biol. Lett. 2018; 14: 20170622. doi: 10.1098/rsbl.2017.0622.
8. Condamine FL, Rolland J, Höhna S, Sperling FA, Sanmartín I. Testing the role of the Red Queen and Court Jester as drivers of the macroevolution of Apollo butterflies. Systematic Biology 2018; 67: 940-964. doi: 10.1093/sysbio/syy009.
9. Gavini SS, Ezcurra C, Aizen MA. Plant–plant interactions promote alpine diversification. Evol. Ecol. 2019; 33: 195-209. doi: 10.1007/s10682-019-09972-5.
10. Balke M, Neven K, Villastrigo A, Ospina-Torres R, Prieto C, et al. Eastern Colombian Páramo *Liodessus* Guignot, 1939 diving beetles are genetically structured, but show signs of hybridization, with description of new species and subspecies (Coleoptera, Dytiscidae). ZooKeys 2023; 1143: 165-187. doi: 10.3897/zookeys.1143.97461.
11. Nilsson AN, HájekJ. (2024): A World Catalogue of the Family Dytiscidae, or the Diving Beetles (Coleoptera, Adephaga). Version 1.I.2024. 2024. Available from: http://waterbeetles.eu
12. Biström O. Generic review of the Bidessini (Coleoptera, Dytiscidae). Contribution to the study of Dytiscidae 37. Acta Zoologica Fennica 1988; 184: 1-41.
13. Balke M, Megna, YS, Zenteno N, Figueroa L, Hendrich L. New *Liodessus* species from the high Andes of Peru (Coleoptera: Dytiscidae, Bidessini). Zootaxa 2020a; 4852: 151-165. doi: 10.11646/zootaxa.4852.2.1.

14. Balke M, Megna YS, Zenteno N, Figueroa L, Hendrich L. Two new species of *Liodessus* Guignot, 1939 diving beetles from northern Peru (Coleoptera, Dytiscidae, Hydroporinae). Alpine Entomology 2020b; 4: 173-178. doi: 10.3897/alpento.4.55139.
15. Balke M, Ospina-Torres R, Suarez-Megna Y, Hendrich L. *Liodessus lacunaviridis* sp. n. from the high Andes of Colombia (Coleoptera: Dytiscidae: Hydroporinae: Bidessini). Tijdschrift voor Entomologie 2020c; 163: 7-11. doi: 10.1163/22119434-20192084.
16. Megna YS, Hendrich L, García-Hernández AL, Ospina-Torres R, Prieto C, Balke M. Diving beetles of the genus *Liodessus* Guignot, 1953 in Colombia, with description of three new species (Coleoptera: Dytiscidae). Aquatic Insects 2019; 40: doi: 10.1080/01650424.2018.1538521.
17. Balke M, Suarez-Megna Y, Ospina-Torres R, Venegas JS, Prieto C, Hendrich L. A new Colombian species of *Liodessus* diving beetles from the Paramo de Sumapaz (Coleoptera, Dytiscidae, Bidessini). ZooKeys 2021; 1059: 79-87. doi: 10.3897/zookeys.1059.70134.
18. Ratnasingham S, Hebert PD. BOLD: The Barcode of Life Data System (www.barcodinglife.org). Mol. Ecol. Notes 2007; 7: 355-364. doi: 10.1111/j.1471-8286.2007.01678.x.
19. Peschet R. Mission Géodésique de l'Équateur. Collections recueillis par M. le Dr Rivet (Coléoptères: Dytiscides). Bulletin du Muséum National d'Histoire Naturelle Paris 1923; 29: 61-63.
20. Kearse M, Moir R, Wilson A, Stones-Havas S, Cheug M, Sturrock S, et al. Geneious Basic: an integrated and extendable desktop software platform for the organization and analysis of sequence data. Bioinformatics 2023; 28: 1647-1649. doi: 10.1093/bioinformatics/bts199.
21. Katoh K, Standley DM. MAFFT multiple sequence alignment software version 7: improvements in performance and usability. Mol. Biol. Evol. 2013; 30: 772-780. doi: 10.1093/molbev/mst010.

22. Stephens M, Smith NJ, Donnelly P. A new statistical method for haplotype reconstruction from population data. American Journal of Human Genetics 2001; 68: 978-989. doi: 10.1086/319501 (2001).

23. Clement M, Posada D, Crandall KA. TCS: A computer program to estimate gene genealogies. Mol. Ecol. 2000; 9: 1657-1659. doi: 10.1046/j.1365-294x.2000.01020.x.

24. Santos AM dos, Cabezas MA, Tavares AI, Xavier R, Branco M. tcsBU: a tool to extend TCS network layout and visualization. Bioinformatics 2016; 32: 627-628. doi: 10.1093/bioinformatics/btv636.

25. Rozas J, Ferrer-Mata A, Sánchez-DelBarrio JC, Guiraro-Rico S, Librado P, Ramos-Onsins SE, et al. DnaSP 6: DNA sequence polymorphism analysis of large datasets. Molecular Biology and Evolution 2017; 34: 3299-3302. doi: 10.1093/molbev/msx248.

26. Van Rossum G, Drake FL. Python 3 Reference Manual. Scotts Valley, CA: CreateSpace; 2009.

27. Waskom ML. Seaborn: statistical data visualization. Journal of Open Source Software 2021; 6: 3021. 10.21105/joss.03021.

28. ICZN. International Code of Zoological Nomenclature Fourth Edition. International Trust for Zoological Nomenclature, Natural History Museum, London; 1999.

29. Laurière C. Paul Rivet. Le savant et le politique. Paris, Publications scientifiques du Muséum national d’histoire naturelle, 2008.

30. Calpa-Anaguano EV, Graham CH, da Silva FR. Pleistocene climate oscillations and habitat connectivity contributed to avian beta-diversity in the megadiverse Colombian Paramo ecosystems. Journal of Biogeography 2021; 48: 3004-3015. doi: 10.1111/jbi.14251.

31. Finn DS, Encalada AC, Hampel H. Genetic isolation among mountains but not between stream types in a tropical high-altitude mayfly. Freshwater Biology 2016; 61: 702-714. doi: 10.1111/fwb.12740.

32. Jong MA de, Wahlberg N, Eijk M van, Brakefield PM, Zwaan BJ. Mitochondrial DNA signature for range-wide populations of *Bicyclus anynana* suggests a rapid expansion from recent refugia. PloS One 2011; 6: e21385. doi: 10.1371/journal.pone.0021385.
33. Zhan H-X, Hao Z-P, Tang R, Zhou J-J, Hou S-M. High genetic diversity and strong genetic structure of Strongyllodes variegatus populations in oilseed rape production areas of China. BMC Ecology and Evolution 2021; 21: 18. doi: 10.1186/s12862-021-01752-6.
34. Knowlton, N. Sibling species in the sea. Annu. Rev. Ecol. Syst. 1993; 24: 189-216.
35. Reidenbach K R, Neafsey DE, Costantini C, Sagnon N, Simard F, Ragland GJ, Egan SP, et al. Patterns of genomic differentiation between ecologically differentiated M and S forms of Anopheles gambiae in West and Central Africa. Gen. Biol. Evol. 2012; **4**: 1202-1212. doi: 10.1093/gbe/evs095.
36. Struck TH, Cerca J. Cryptic Species and Their Evolutionary Significance. eLS. John Wiley & Sons, Ltd: Chichester. 2019. doi: 10.1002/9780470015902.a0028292.
37. Olivares I, Tusso S, Sanín MJ, La Harpe M de, Loiseau O, Rolland J, et al. Hyper-Cryptic radiation of a tropical montane plant lineage. Mol. Phyl. Evol. 2024; 190: 107954. doi: 10.1016/j.ympev.2023.107954.
38. Ganopolski A, Winkelmann R, Schellnhuber H. Critical insolation–CO_2 relation for diagnosing past and future glacial inception. Nature 2016; 529: 200-203; doi: 10.1038/nature16494.

Acknowledgements

This work was made possible by a grant from the Alexander von Humboldt foundation under the Research Group Linkage Program (Evolution of the high Andean insect fauna project) which enabled the Colombian and German teams to closely work together and develop new partnerships on both sides.

We are grateful for the generous support from the SNSB-Innovative scheme, funded by the Bayerisches Staatsministerium für Wissenschaft und Kunst (Project: “Geographische Isolation, Endemismus und Artbildungsprozesse bei Insekten in der hochmontanen Páramo Kolumbiens (und darüber hinaus)”).

Consortium of European Taxonomic Facilities (CETAF) data use statement: “Data on genetic material contained in this taxonomic article are published for non-commercial use only. Utilization by third parties for purposes other than non-commercial scientific research may infringe the conditions under which the genetic resources were originally accessed, and should not be undertaken without obtaining consent from the original provider of the genetic material.”

The international cooperations underlying this project were formalized with MoUs, based on which we acquired all necessary collecting, export and DNA sequencing permits. These are archived at SNSB-ZSM and available upon request from Michael Balke. The material collected in Ecuador is supported by the permit “Diversity distribution and evolution of the beetles of Ecuador - multidisciplinary approach with emphasis on systematics genetics and biogeography” (MAATE-DBI-CM--2022-0255) issued by the Ministry of Environment, Water and Ecological Transition of Ecuador.

Data availability statement

The data presented here have been deposited at Dryad (datadryad.org). The locality as well as sequence data are also available in the Barcode of Life Data System (“BOLD”, https://www.boldsystems.org).

Additional Information

Competing Interests: The authors declare no competing interests.

Figure legends

Figure 1. Neighbour-joining tree for the sublineages of *Liodessus quillacinga*. **Green** *L. quillacinga ecuadoriensis* ssp. nov., **Blue** *L. quillacinga cochaensis*, **Pink** *L. quillacinga quillacinga*, **Red** *L. quillacinga cumbalis*, **Black** outgroup.

Figure 2. Maps of **A** sampling localities and **B** lineages of *Liodessus quillacinga*. Blue coloured areas represent Páramo habitats.

Figure 3. Habitus of **A** *L. quillacinga quillacinga* holotype (HT) and paratype (PT), **B** *L. quillacinga cochaensis* holotype (HT) and paratype (PT), **C** *L. cumbalis* holotype (HT) and paratype (PT), **D–F** *L. quillacinga ecuadoriensis* ssp. nov. paratypes from different localities. Scale bar: 1 mm.

Figure 4. Median lobes in lateral view of *Liodessus* species **A** *L. quillacinga quillacinga* holotype (HT) and paratype (PT), **B** *L. quillacinga cochaensis* holotype (HT) and paratype (PT), **C** *L. cumbalis* holotype (HT) and **D–G** *L. quillacinga ecuadoriensis* ssp. nov. paratypes from different localities. Scale bar: 0.3 mm.

Figure 5. Median lobes in ventral view of *Liodessus* species **A** *L. quillacinga cumbalis* holotype (HT), **B** *L. quillacinga quillacinga* holotype (HT) and paratype (PT), **B** *L. quillacinga cochaensis* holotype (HT) and paratype (PT), **D–H** *L. quillacinga ecuadoriensis* ssp. nov. paratypes from different localities. Scale bar: 0.1 mm.

Figure 6. Haplotype network for *Liodessus quillacinga*, including information of main sublineages.

Figure 7. Heatmap of pairwise comparison of Fst values among *L. quillacinga* sublineages.

Figure 8. Holotype of *Liodessus quillacinga ecuadoriensis* ssp. nov. from the Laguna San Pablo, 2,700 m, 0.199° -78.2331°. Scale bar: 1 mm.

Figure 9. **A–D** Habitus of *Liodessus quillacinga ecuadoriensis* ssp. nov. paratypes from different localities.

Figure 10. Habitats of *Liodessus quillacinga ecuadoriensis* ssp. nov., **A** Otavalo, Laguna San Pablo (locus typicus); **B** Mt. Cotopaxi, small wet patch; **C** Mt. Antisana, La Mica area.

Figure 11. Neighbour-joining tree for the sublineages of *Liodessus quimbaya*. **Blue** *L. quimbaya azufralis* stat. nov., **Green** *L. quimbaya quimbaya*, **Pink** *L. quimbaya paletara* ssp. nov., **Black** outgroup.

Figure 12. Maps of **A** sampling localities and **B** lineages of *Liodessus quimbaya*. Blue coloured areas represent Páramo habitats.

Figure 13. Habitus of **A** *L. quimbaya azufralis* stat. nov. paratypes (PT), **B** *L. quimbaya quimbaya* holotype (HT), **C–D** *L. quimbaya azufralis* stat. nov. from different localities. Scale bar: 1 mm.

Figure 14. Habitus of **A** *L. quimbaya quimbaya* from Malvaza, **B** *L. quimbaya paletara* ssp. nov. paratypes and holotype (HT), **C–D** *L. quimbaya azufralis* stat. nov. from different localities. Scale bar: 1 mm.

Figure 15. Median lobes in lateral view of **A** *L. quimbaya azufralis* stat. nov. holotype (HT) and paratype (PT), **B–D** from Ecuador; **E** *L. quimbaya quimbaya* holotype (HT) and paratype (PT), **F** *L. quimbaya paletara* ssp. nov. Scale bar: 0.3 mm.

Figure 16. Median lobes in lateral view of *Liodessus* species **A** *L. quimbaya paletara* ssp. nov. paratype and holotype (HT), **B** *L. quimbaya quimbaya* from Malvaza. Scale bar: 0.3 mm.

Figure 17. Median lobes in ventral view of *Liodessus* species **A** *L. quimbaya quimbaya* holotype (HT) and paratype (PT), **B** *L. quimbaya azufralis* stat. nov. holotype (HT) and paratype (PT), **C** *L. quimbaya paletara* ssp. nov. holotype (HT) and paratype (PT), **D** *L. quimbaya quimbaya* from Malavaza, **E–I** *L. quimbaya azufralis* stat. nov. from different localities. Scale bar: 0.1 mm.

Figure 18. Haplotype network for *Liodessus quimbaya*, including information of main sublineages.

Figure 19. Heatmap of pairwise comparison of Fst values among *L. quimbaya* sublineages.

Figure 1

Click here to access/download;Figure;Figure_1.pdf

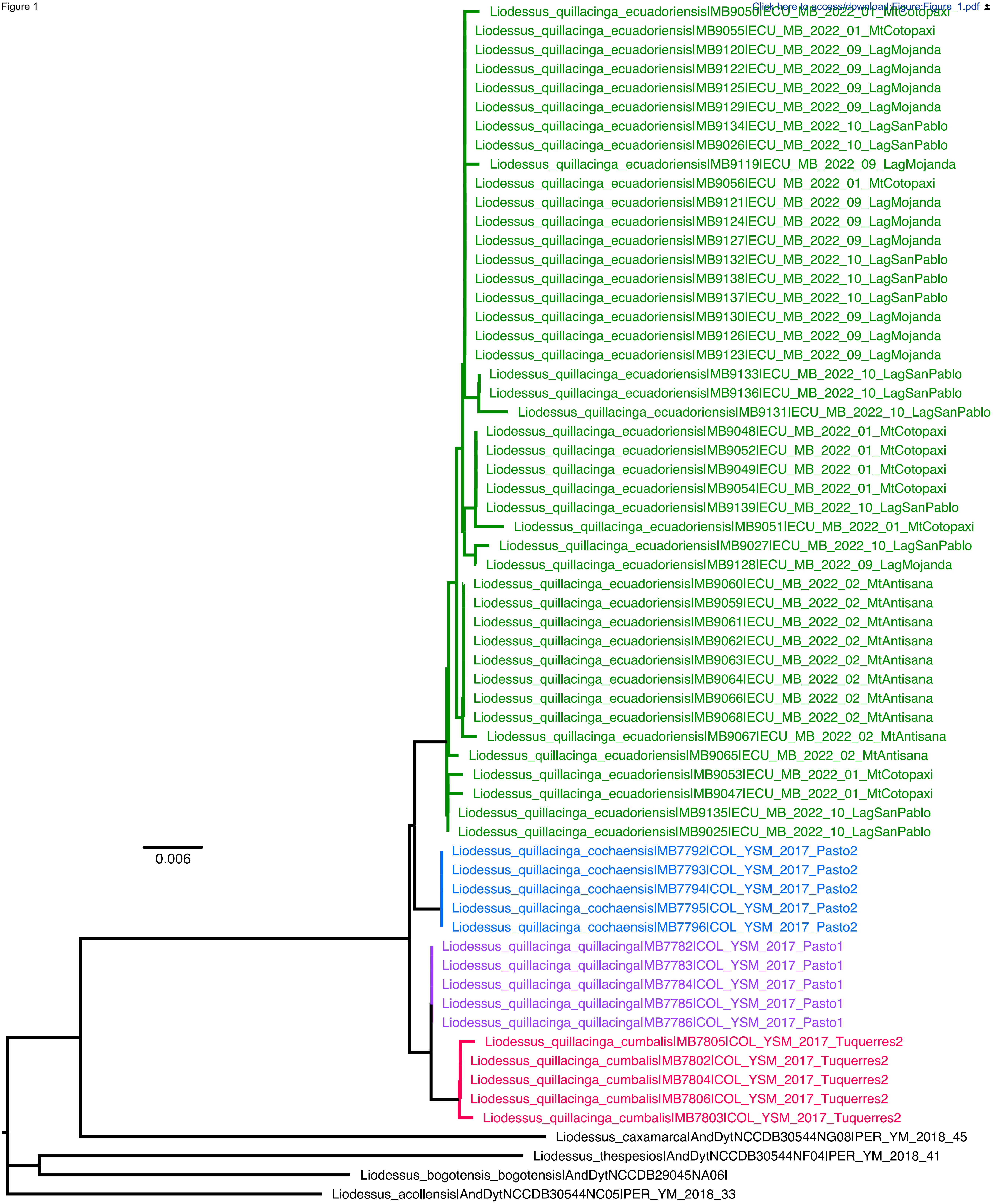

Figure 2

Click here to access/download;Figure;Figure_2.png

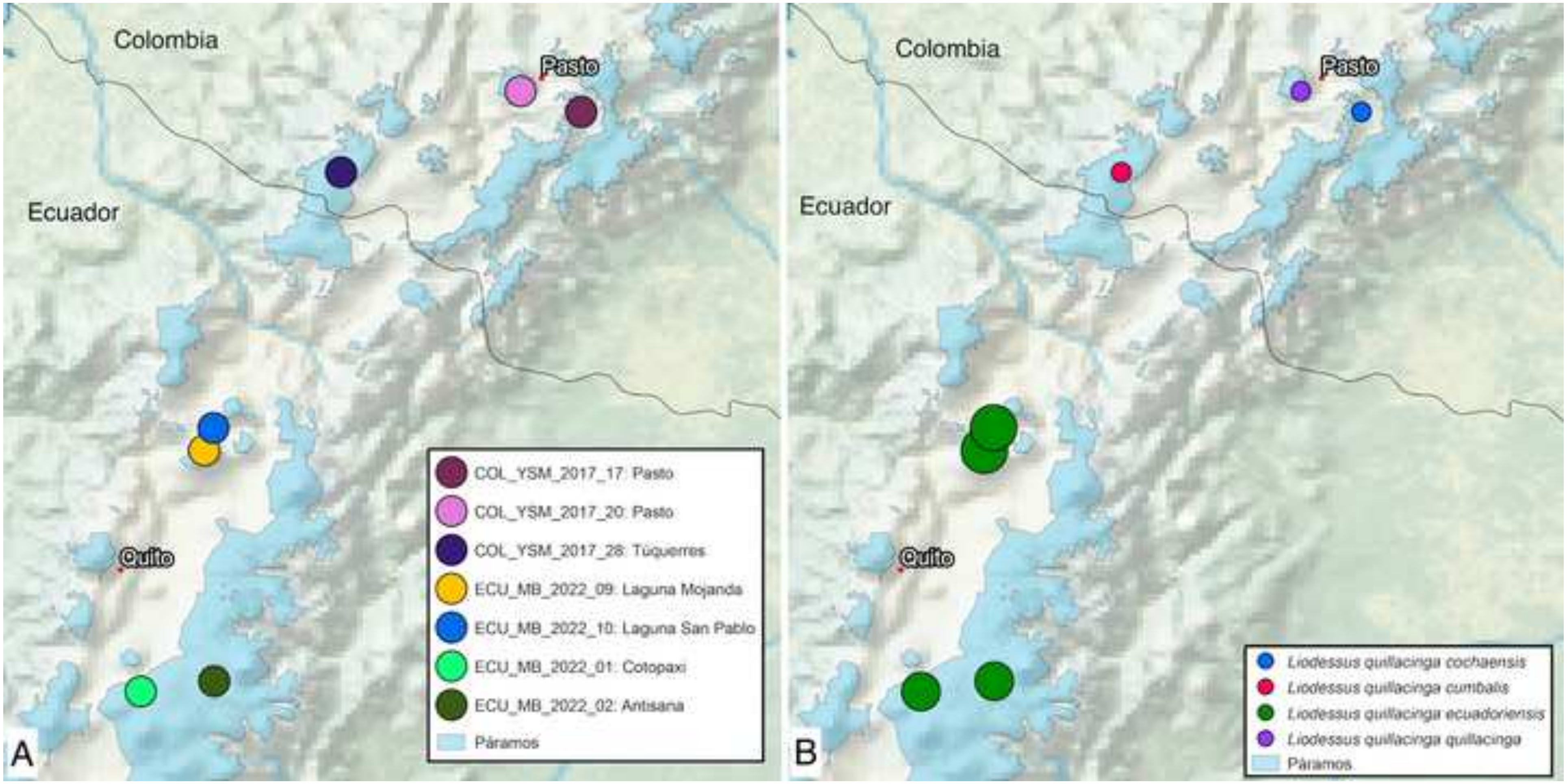

Figure 3

Click here to access/download;Figure;Figure_3.png

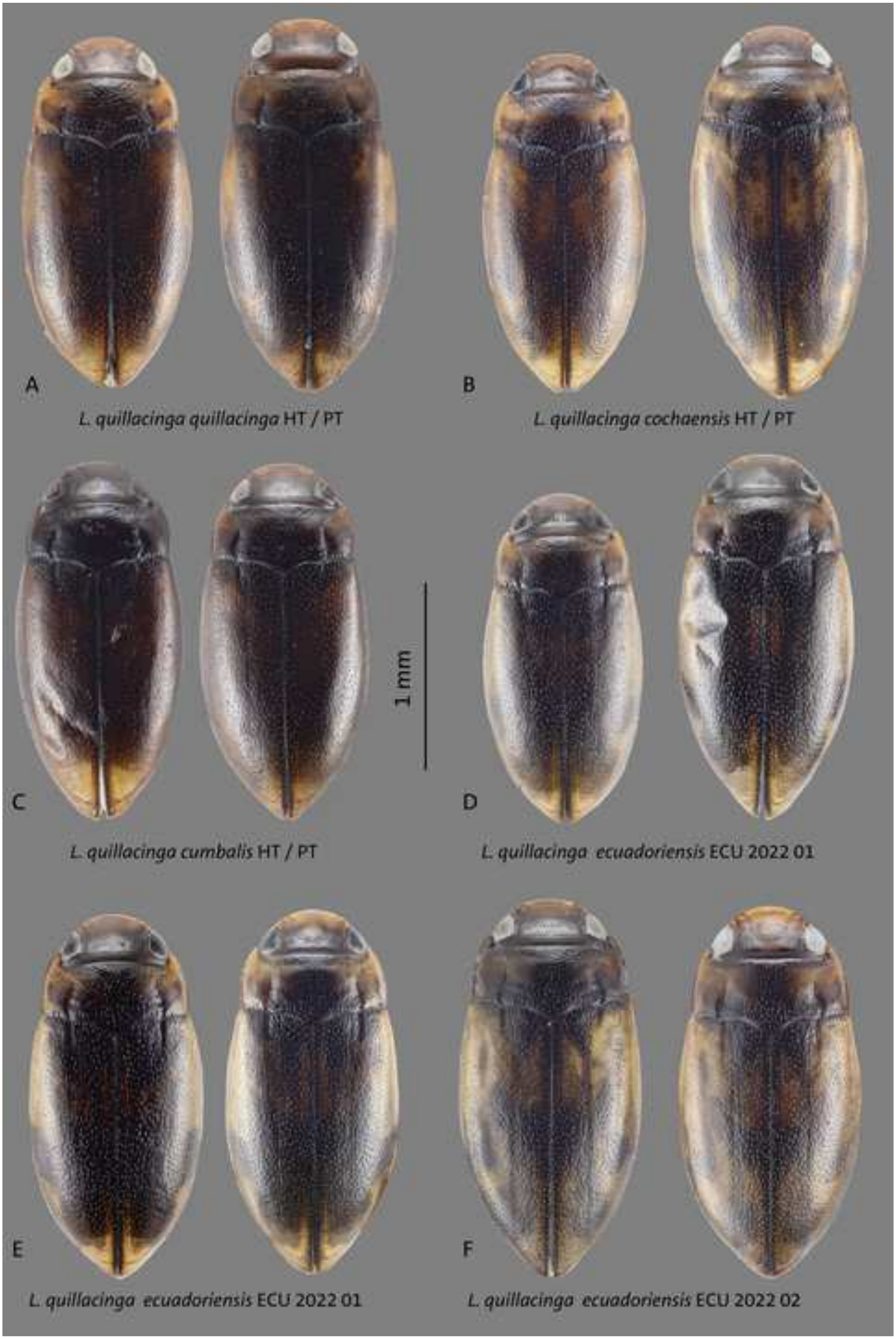

Click here to access/download;Figure;Figure_4.png

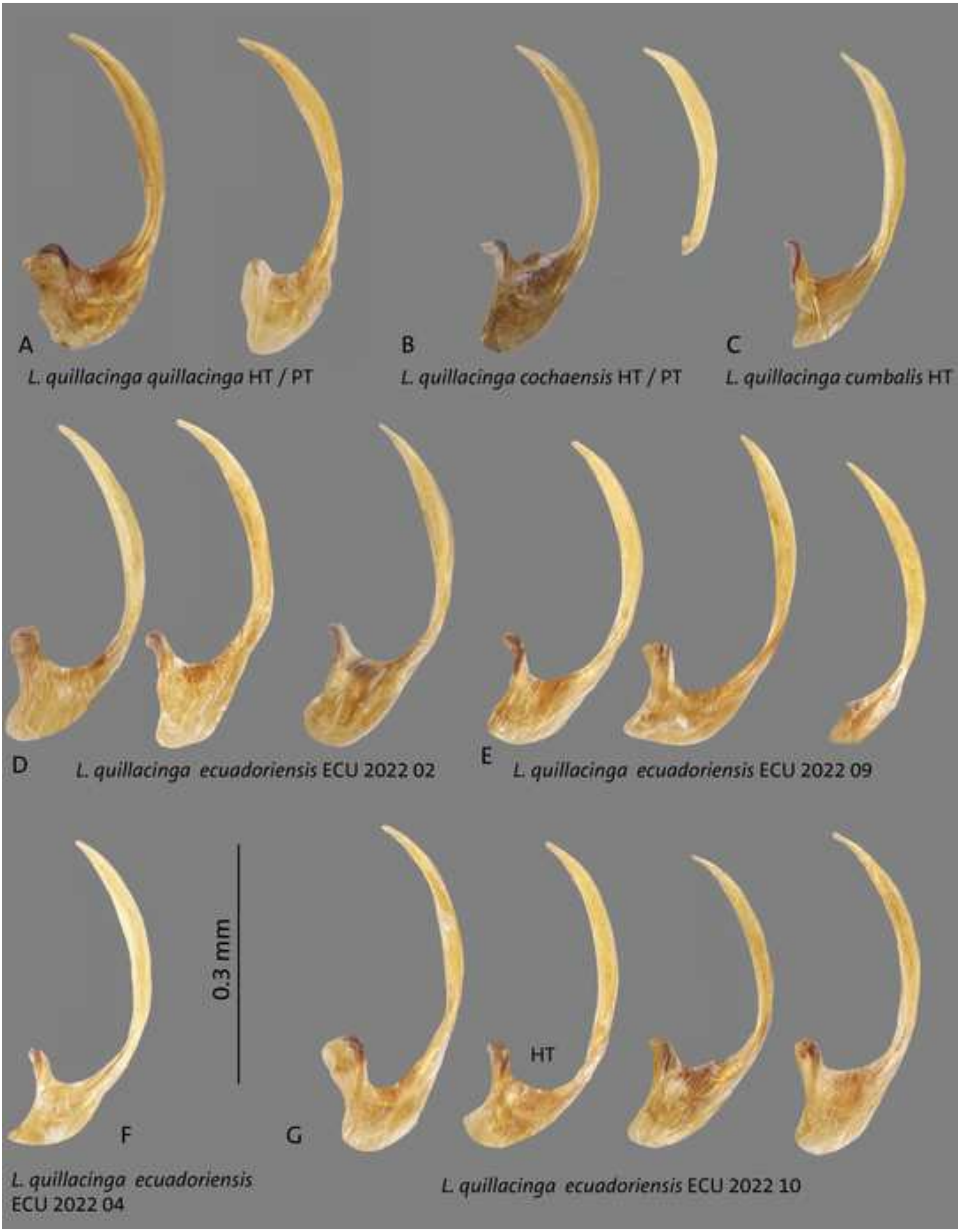

Figure 5

Click here to access/download;Figure;Figure_5.png

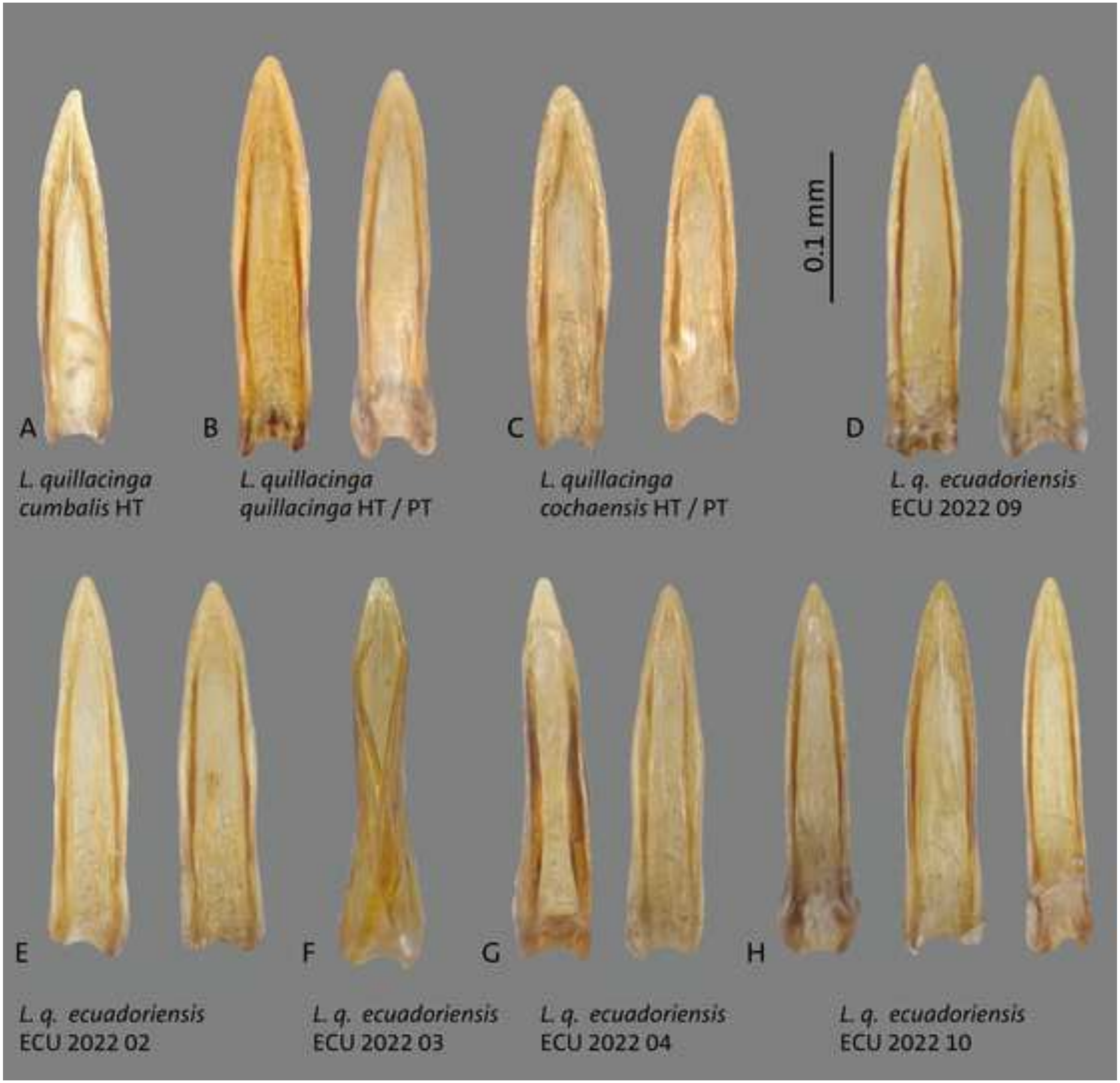

Figure 6

Click here to access/download;Figure;Figure_6.png

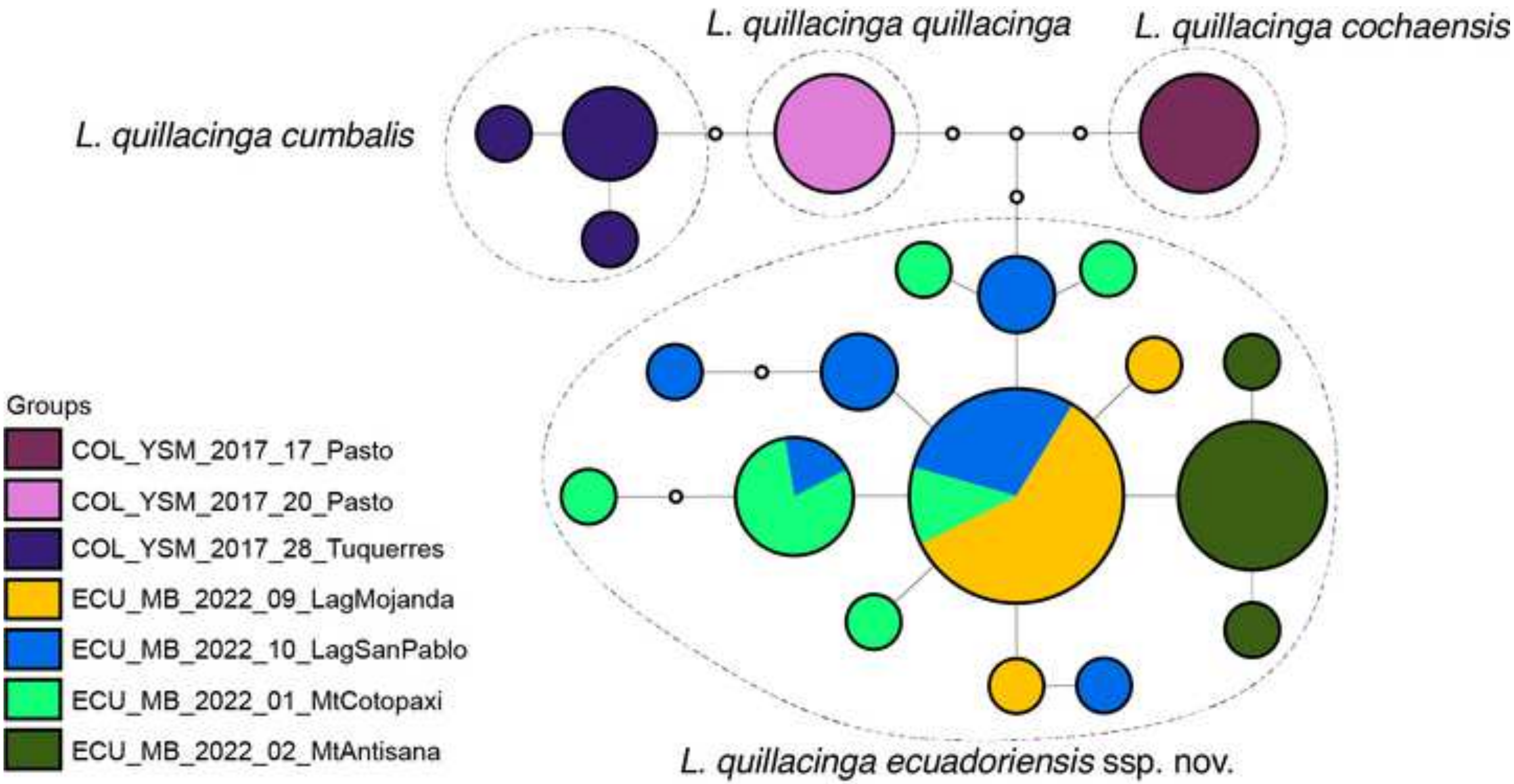

Figure 7

Click here to access/download;Figure;Figure_7.pdf

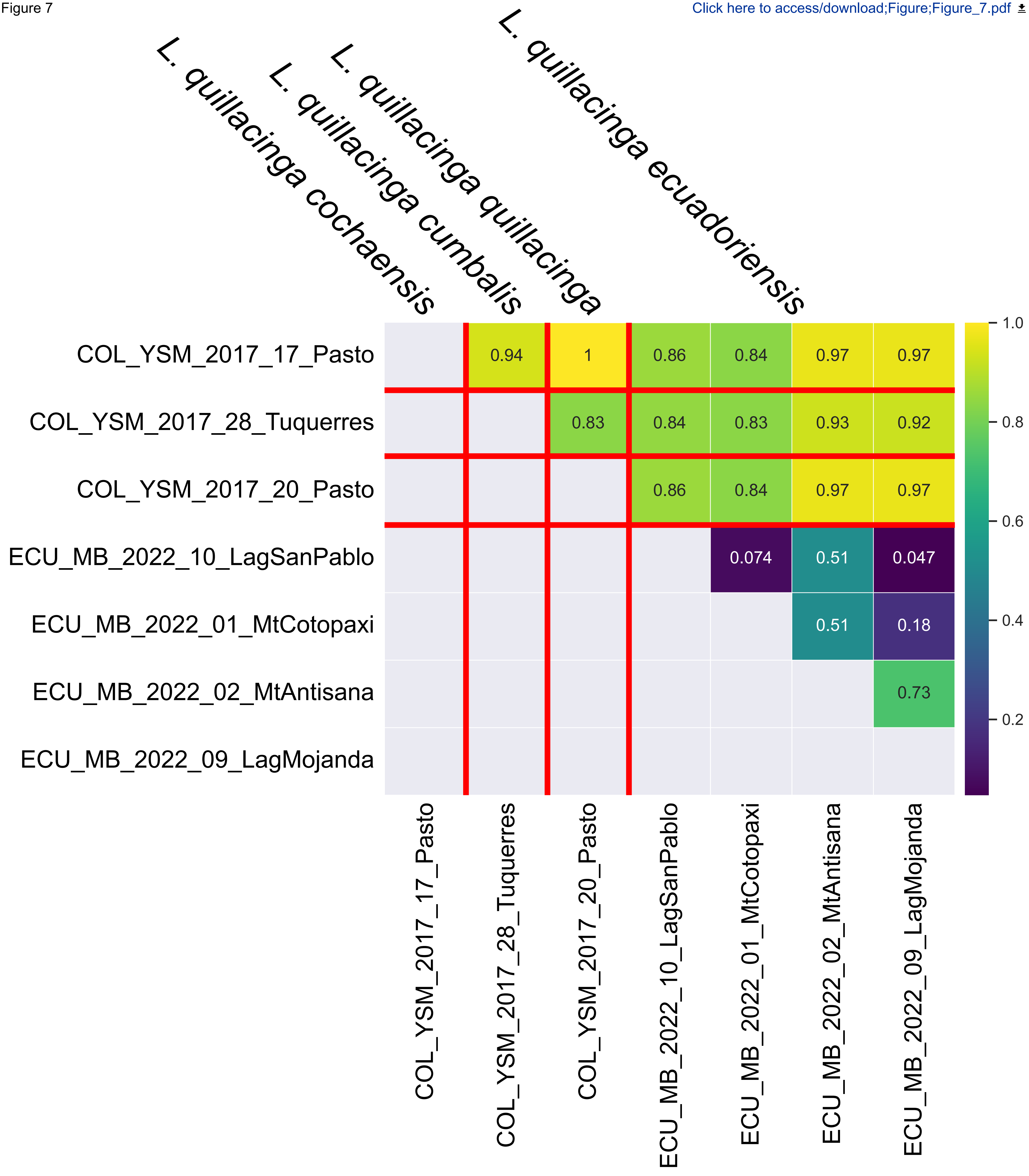

Figure 8

Click here to access/download;Figure;Figure_8.png

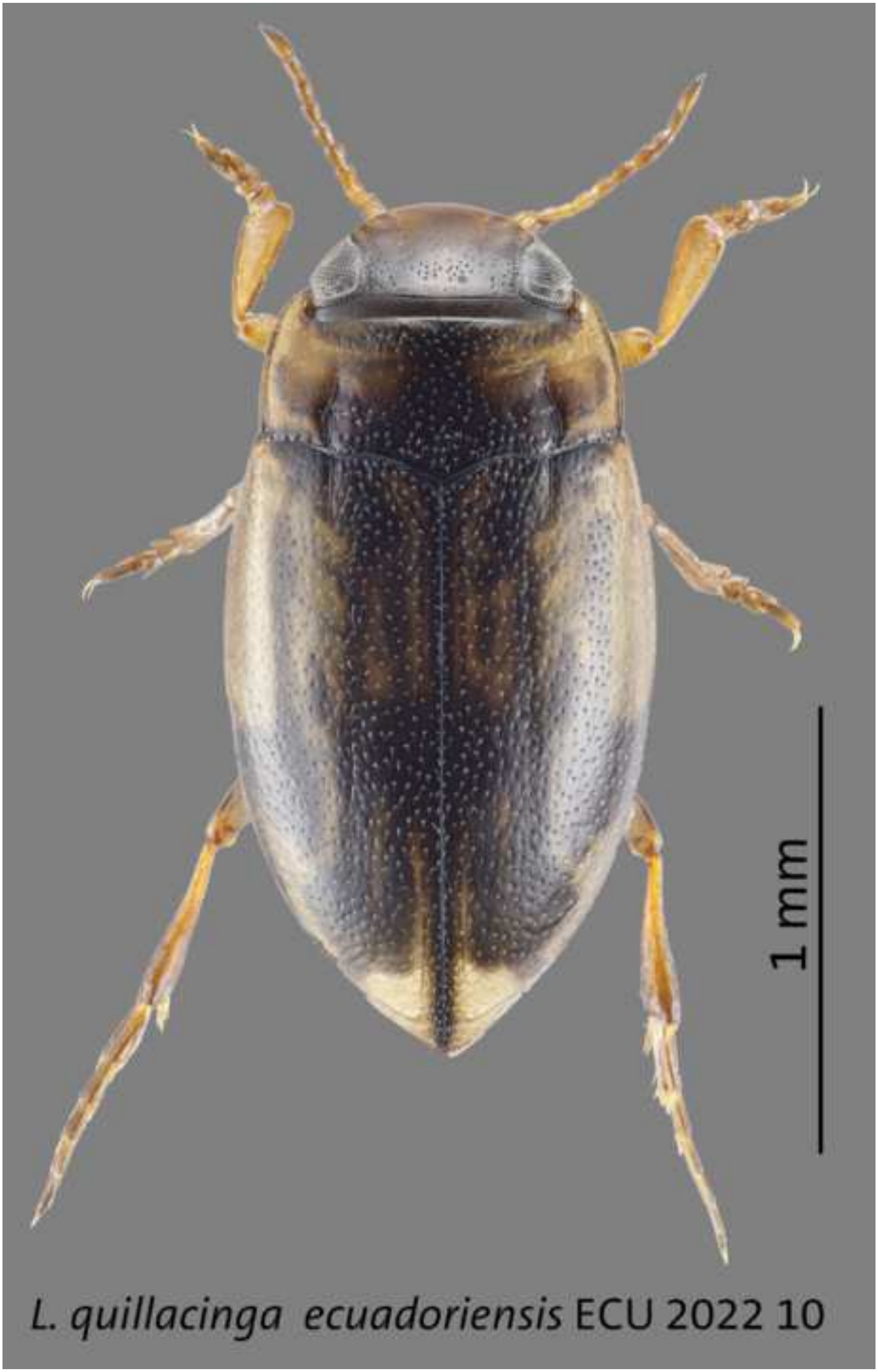

Click here to access/download;Figure;Figure_9.png

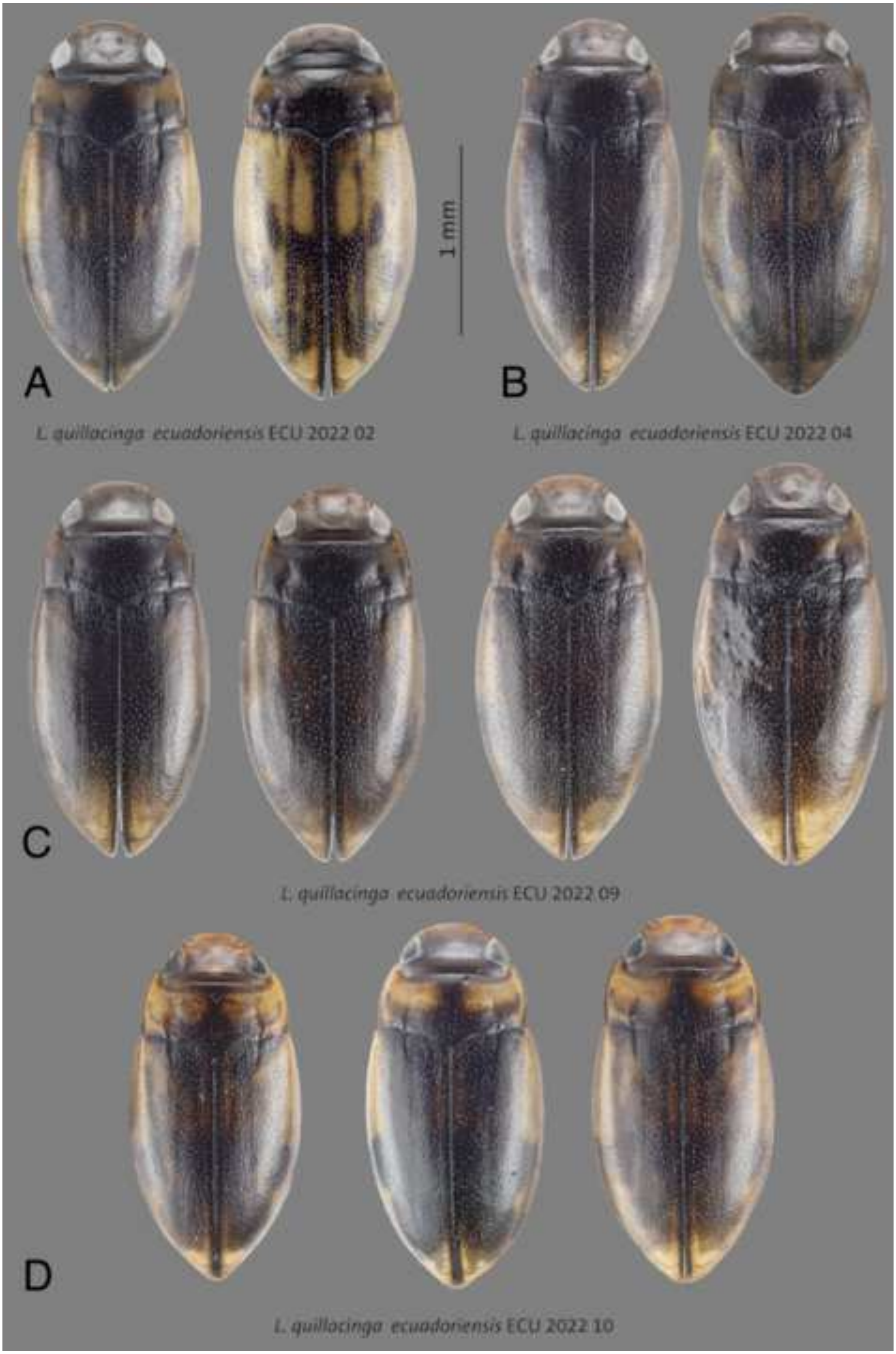

Figure 10

Click here to access/download;Figure;Figure_10.png

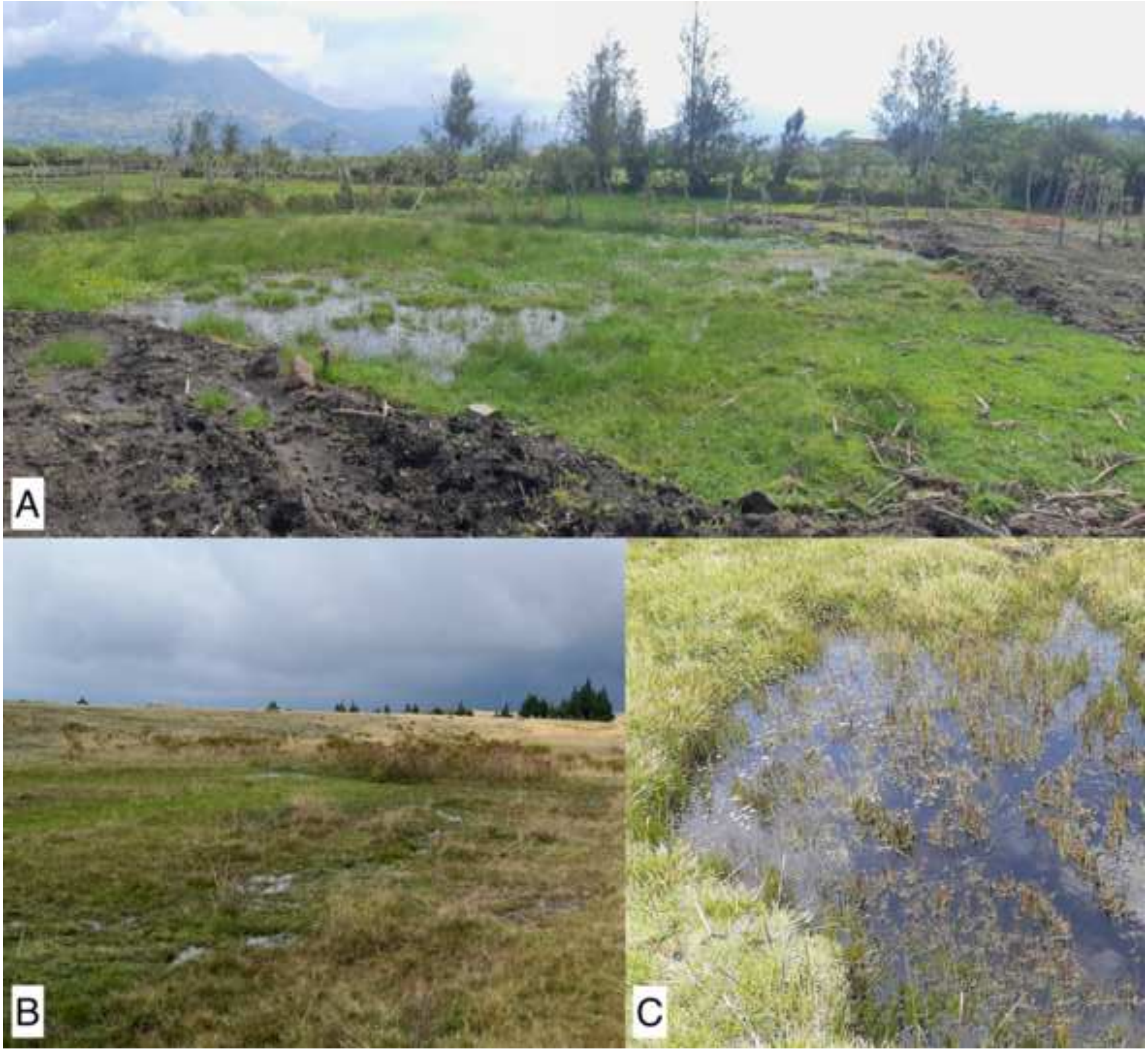

Figure 11

Click here to access/download;Figure;Figure_11.pdf

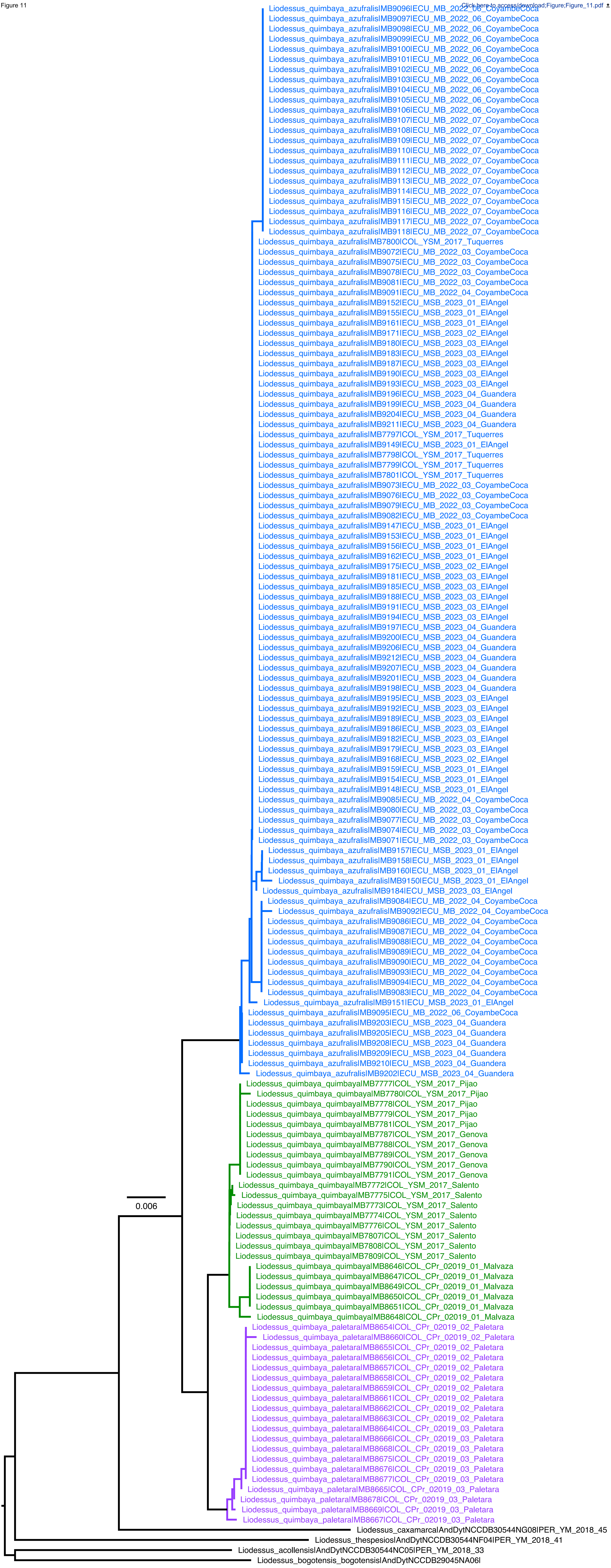

Figure 12

Click here to access/download;Figure;Figure_12.png

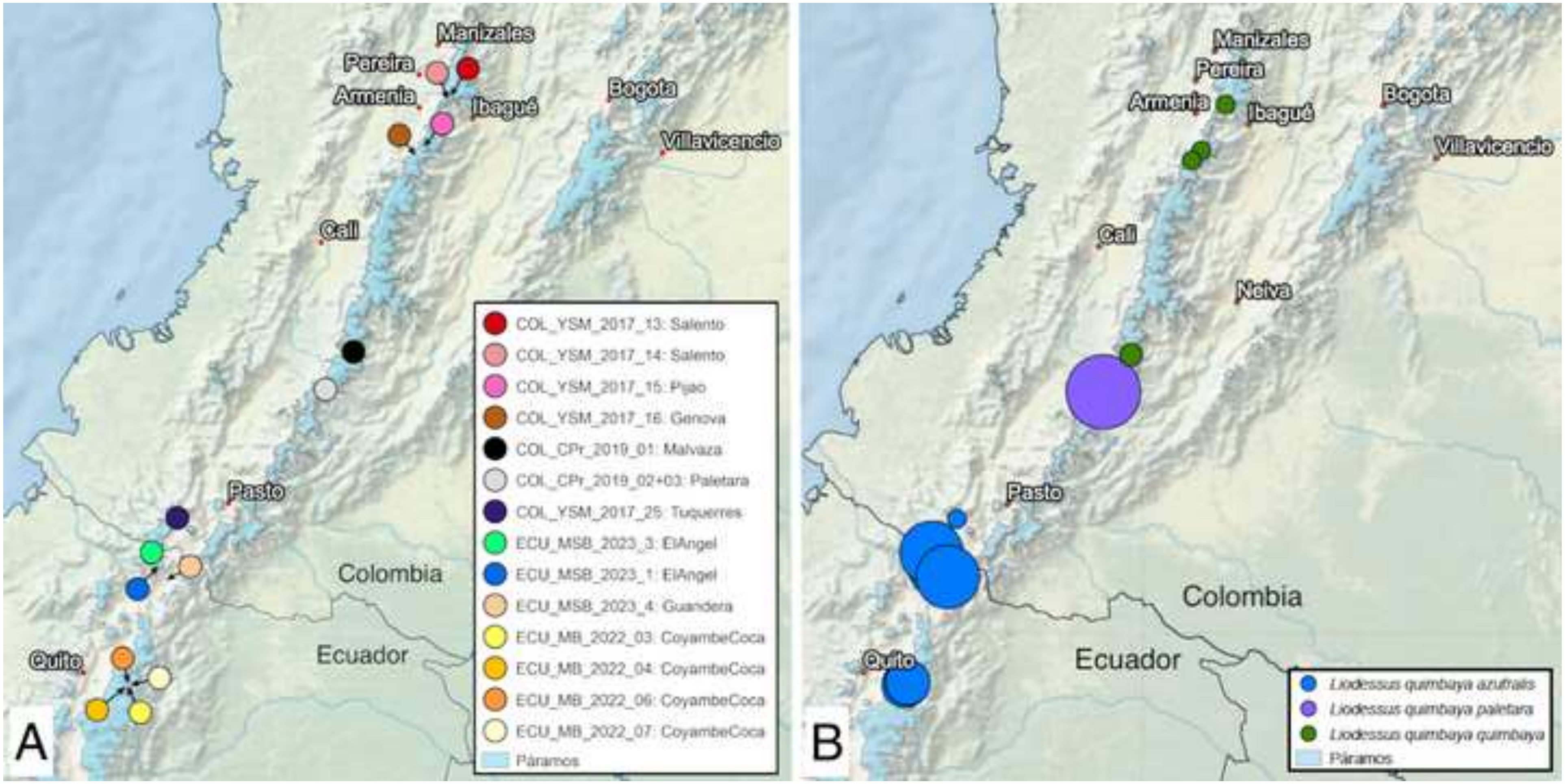

Figure 13

Click here to access/download;Figure;Figure_13.png

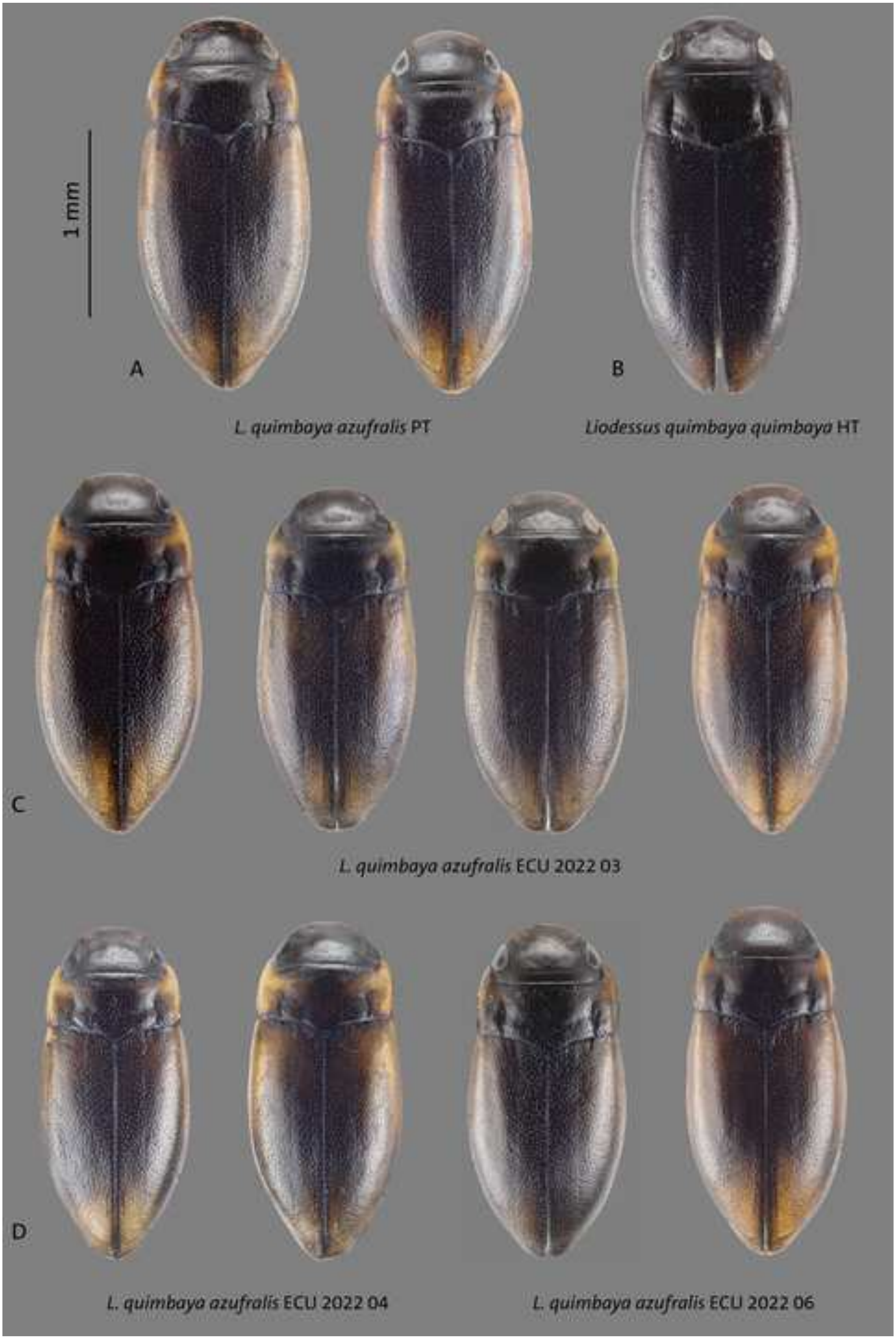

Figure 14

Click here to access/download;Figure;Figure_14.png

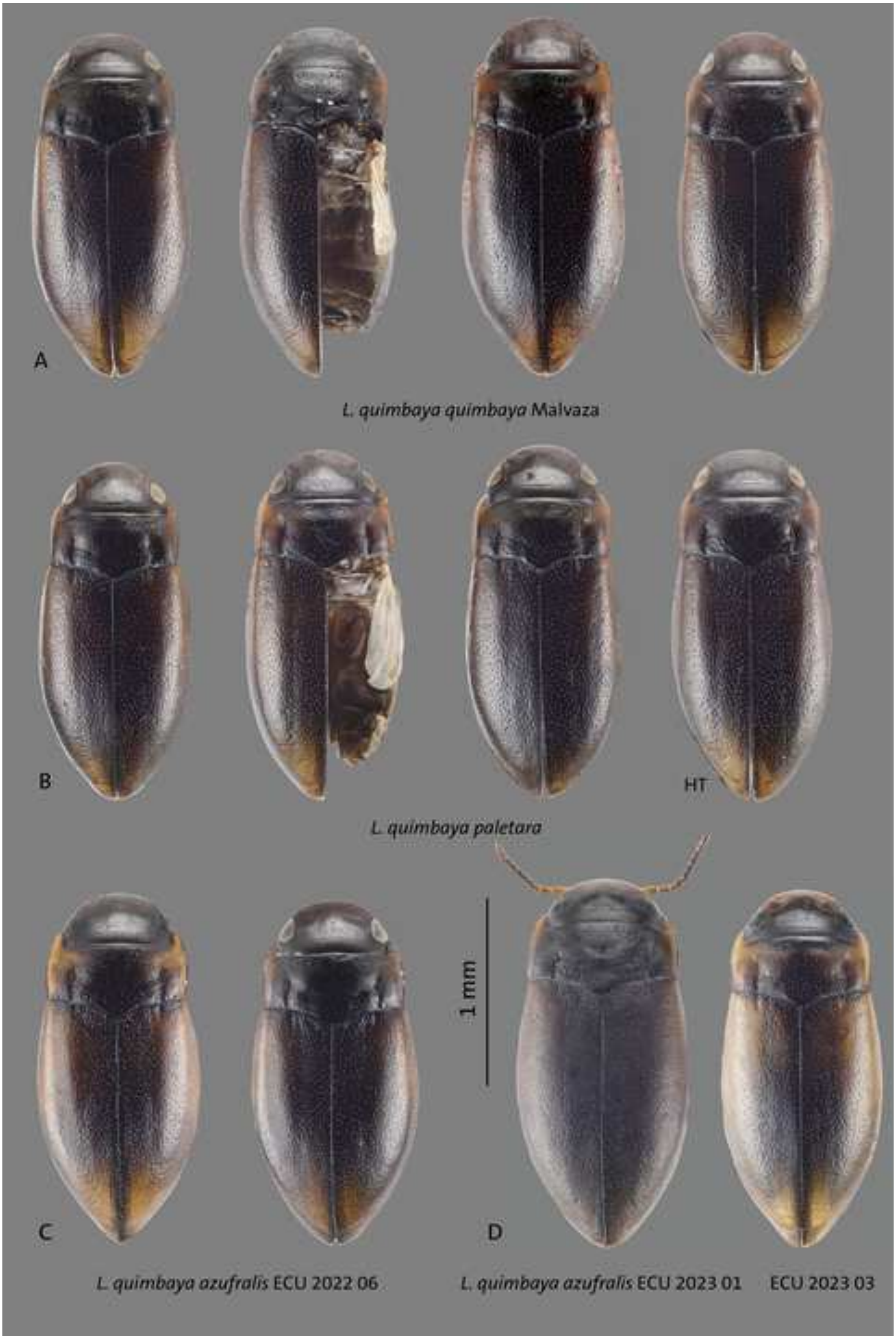

Figure 15 Click here to access/download;Figure;Figure_15.png

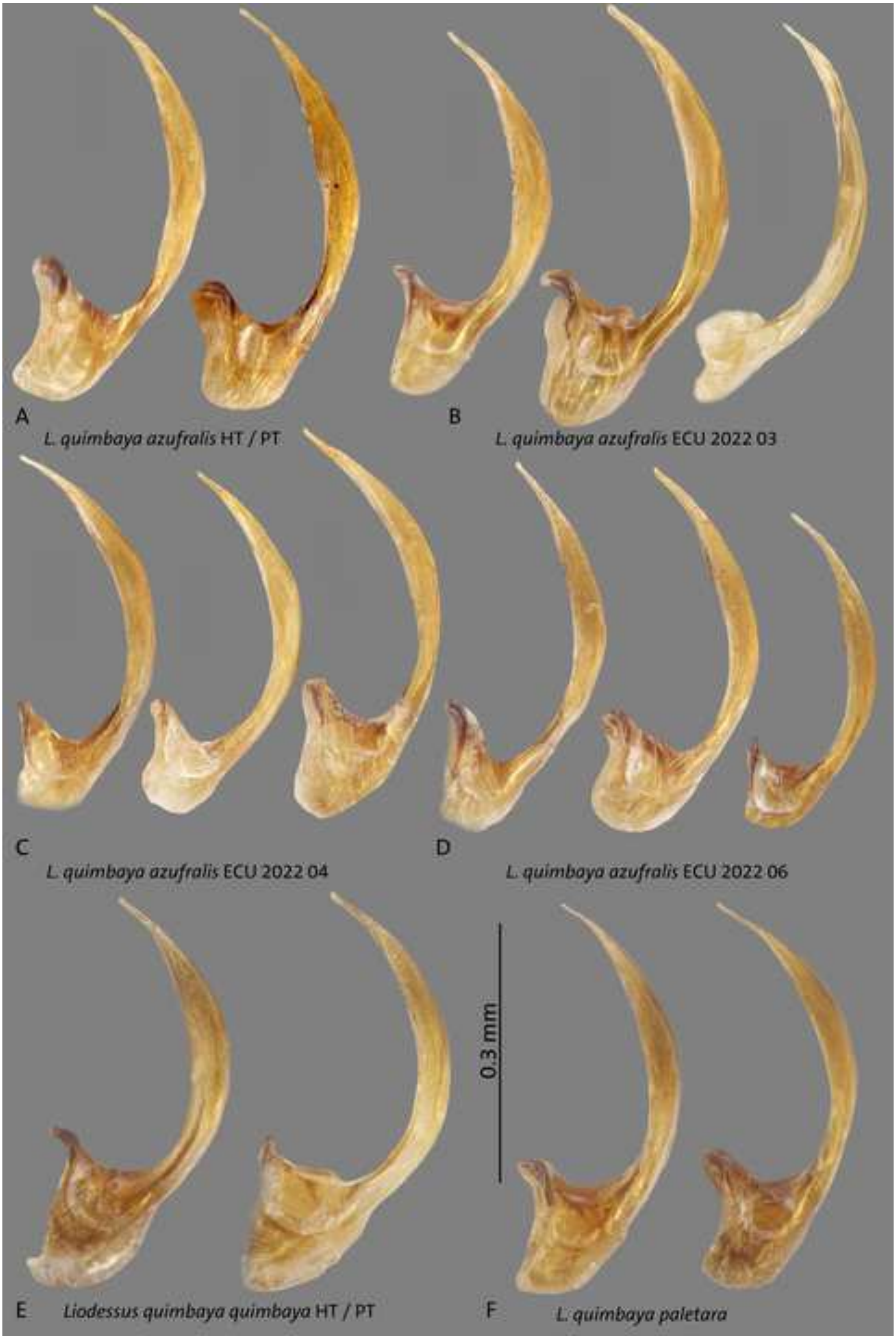

Figure 16 Click here to access/download;Figure;Figure_16.png

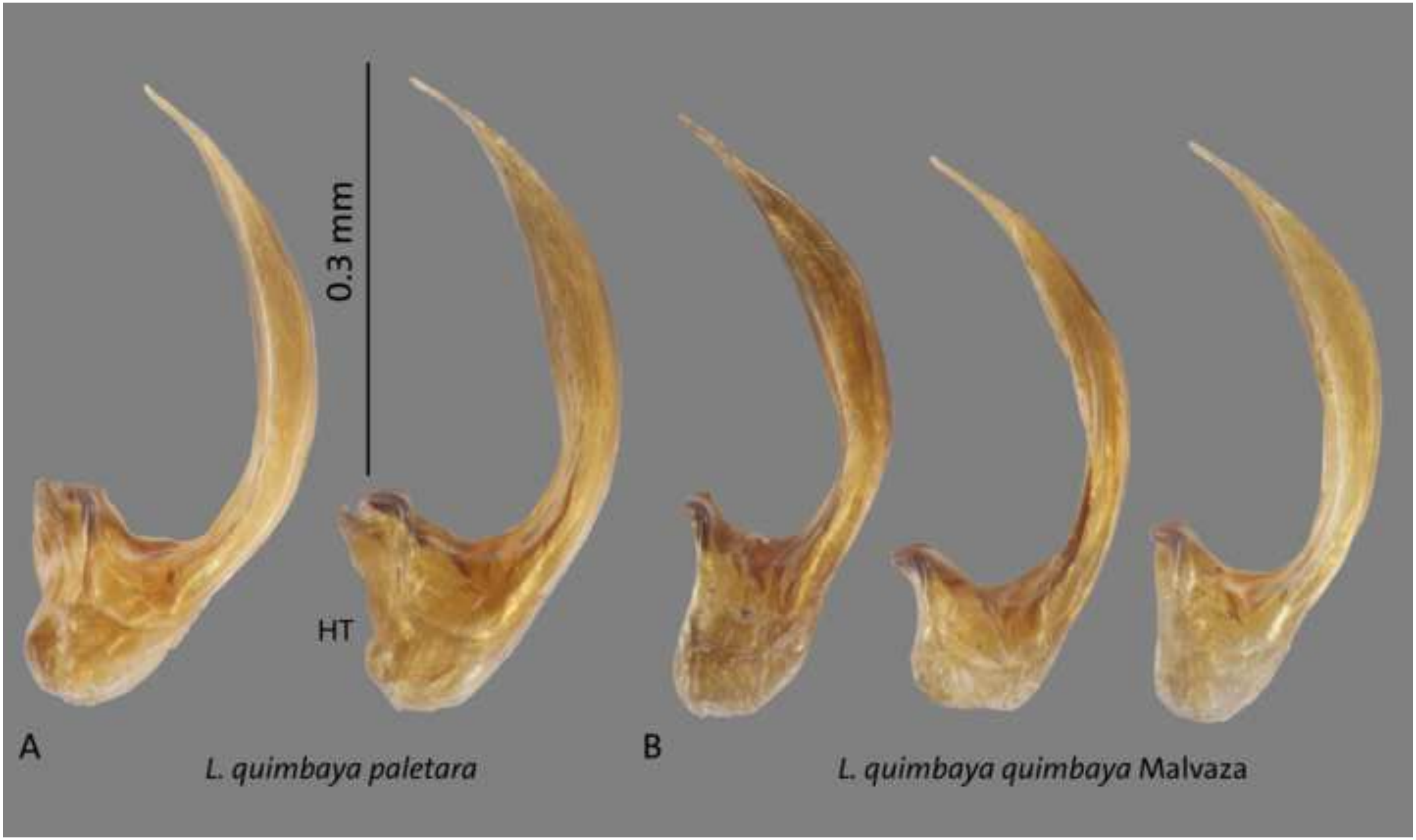

Figure 17

Click here to access/download;Figure;Figure_17.png

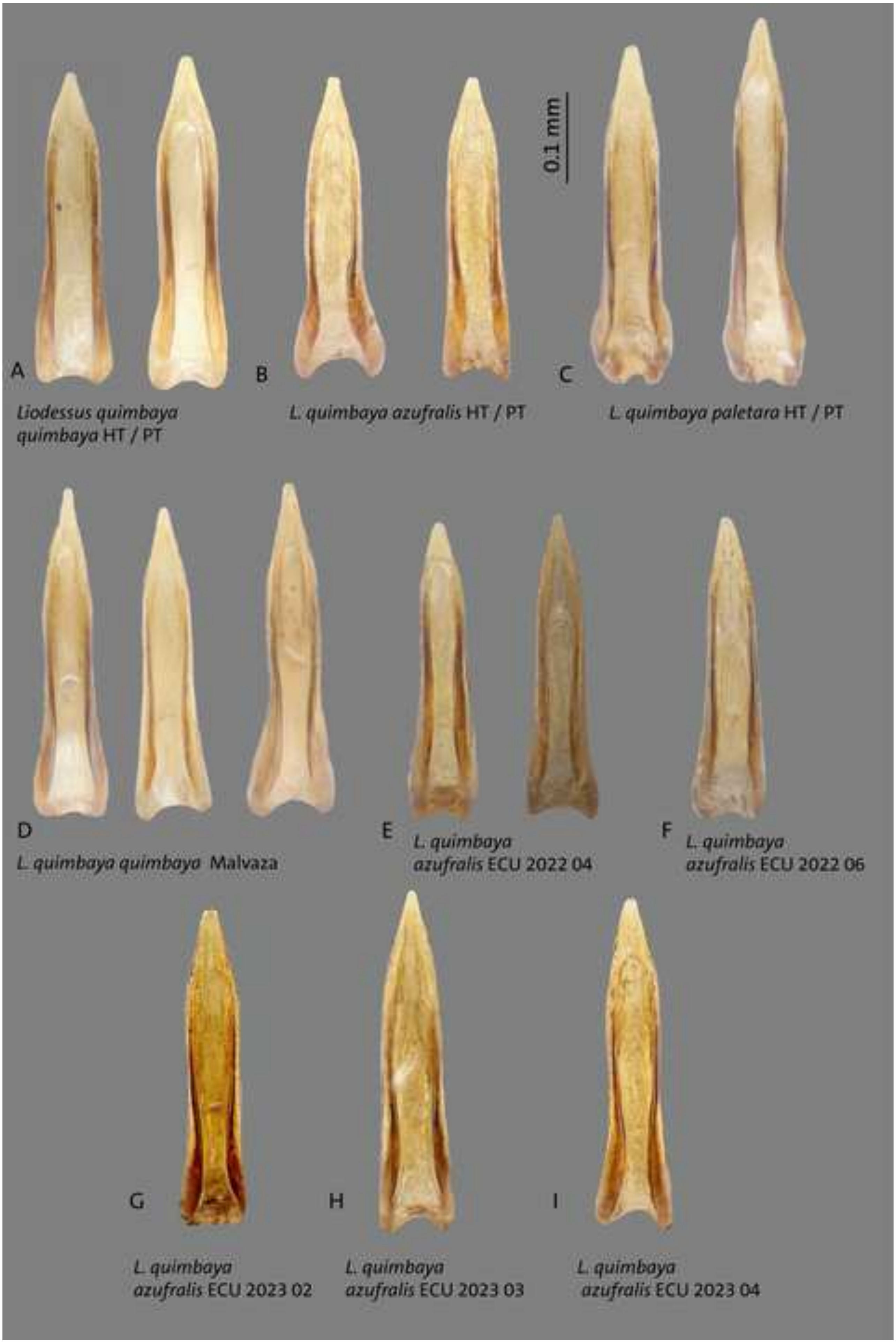

Figure 18

Click here to access/download;Figure;Figure 1

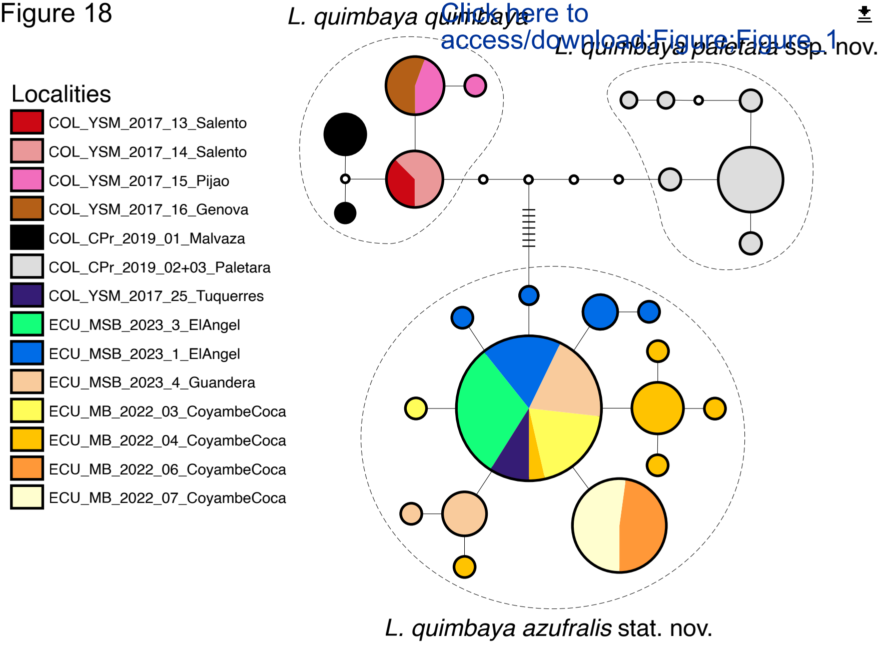

Figure 19

Click here to access/download;Figure;Figure_19.pdf

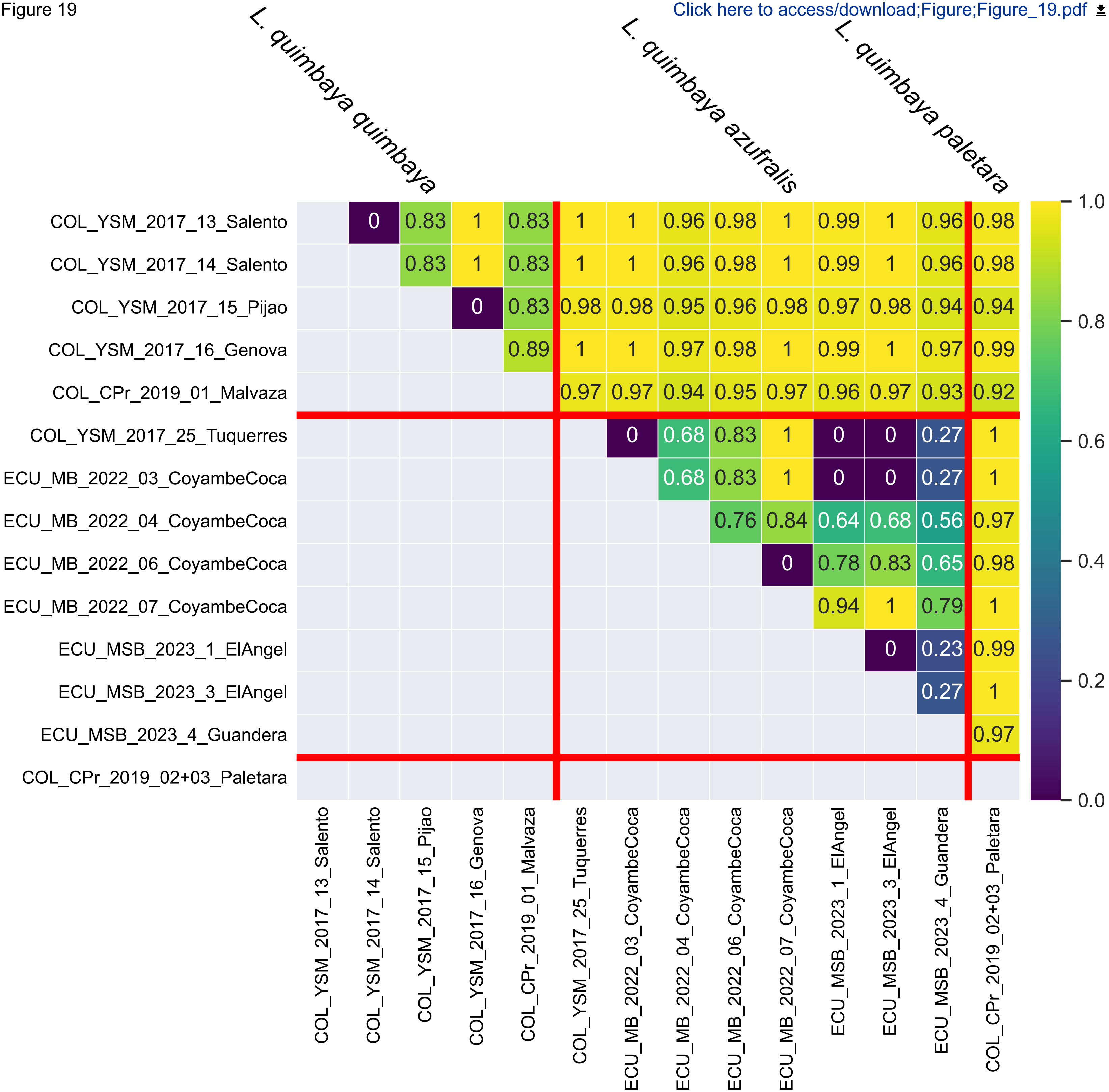

Supplement: Attachment — Submitted filename: PONE-D-24-13211_reviewerSpecificComments.pdf [file pone.0308683.s003.pdf]
