# Supplementary material for: Recent range expansion and lineage idiosyncratic population structure of Liodessus diving beetles in the high Andes (Coleoptera: Dytiscidae, Bidessini)
Source: PLoS One. 2024 Dec 20;19(12):e0308683. doi: 10.1371/journal.pone.0308683 (PMC11661648; doi:10.1371/journal.pone.0308683)
Supplement: Attachment — Submitted filename: Response_letter.docx [file pone.0308683.s005.docx]

Dear Editor,

We are happy to see the positive feedback for our manuscript “Recent range expansion and lineage idiosyncratic population structure of Liodessus diving beetles in the high Andes (Coleoptera: Dytiscidae, Bidessini)”. We have addressed most comments, although some of them are further discussed in this letter. Regarding your recommendation of including some species delimitation methods, we have tested ASAP and PTP, which highlighted some unexpected results in the case of *Liodessus quillacinga* which are not consistent with the other evidence we used. For that reason, we opted to include that information as part of the supplementary material, and added comments in the results and discussion.

Regarding other comments made in your email, please let us clarify some of them:

4. Thank you for stating the following financial disclosure…

**REPLY >>>** The contributions made by Michael Balke were not only funding acquisition, but active work on it (as can be seen as been the first author). We participated in all steps, from field work, to specimen curation, experimental design, data analyses and discussion and manuscript writing. Do you require any specific statement within the financial disclosure?

5. Thank you for stating the following in the Acknowledgments Section of your manuscript…

**REPLY >>>** Thank you for letting us know about our mistake. Indeed, the support of some institutions was not clearly stated in the financial disclosure and was in the acknowledgements. A complete financial disclosure should be used as follows:

“Michael Balke acknowledges support from the Deutsche Forschungsgemeinschaft (BA2152/27-1), project number: 496550039. In the initial phases of this project, Adrián Villastrigo was funded by the Alexander von Humboldt Foundation through a Humboldt Research Fellowship and by the Carl Friedrich von Siemens Foundation at SNSB-ZSM, and this stipend is greatly acknowledged here.

Michael Balke acknowledges support from the EU SYNTHESYS program, projects FR-TAF 6972 and GB-TAF-6776, which supported this research during visits to Natural History Museum in London and Muséum national d’Histoire naturelle in Paris in 2017 to study historical type material.

This project was also generously supported by a grant from the Alexander von Humboldt Foundation under the Research Group Linkage Program (Evolution of the high Andean insect fauna project) which enabled the Colombian and German teams to closely work together and develop new partnerships on both sides. We are also grateful for the generous support from the SNSB-Innovative scheme, funded by the Bayerisches Staatsministerium für Wissenschaft und Kunst (Project: “Geographische Isolation, Endemismus und Artbildungsprozesse bei Insekten in der hochmontanen Páramo Kolumbiens (und darüber hinaus)”).”

**Reviewer #1 >>>** Review | Manuscript: PONE-D-24-13211

SUMMARY AND OVERALL IMPRESSION

The current study presents a novel integrative approach to resolve the taxonomy and population genetic structure using the genus Liodessus Guignot 1939 as a model system. Further, this work explores unique ecosystems, namely Páramo habitats that are good models for investigating population genetic structure due to intermittent geographical isolations and connectivity in the past. Therefore, the work will significantly contribute to advancing the field and will be of interest to the broad community.

**REPLY >>>** Thank you very much for your positive opinion on our research. It is indeed a fantastic study system, and we are confident it will continue to yield great insights into the macro- and microevolutionary factors influencing speciation.

GENERAL COMMENTS

**Reviewer >>>** Overall statistics: In the methods section, it will be helpful from the readers' point of view to describe statistics relevant to analyses performed. Similarly, please describe in detail the statistical results in the results section. It will be useful for replication and repetition of the study.

**REPLY >>>** As you mentioned, we recognize the needs to provide comprehensive methodological details to facilitate replication. We have described all software used and the parameters required for replications (e.g., specific alignment settings, tree-building software with algorithm, haplotype phasing, Fst calculation, …). We believe the information provided in the manuscript is sufficient for replication. The only “missing information” pertains to the visualization of the Fst values using Python 3 and the Seaborn library, which is just a visualization method rather than a proper methodology.

**Reviewer >>>** Population genetic structure: This work includes 147 specimens of L. quimbaya and 59 specimens of L. quillacinga to explore population genetic structure. Which test/tests were used to check data adequacy prior to analysis?

**REPLY >>>** As stated in the methodology (lines 145 to 163), we followed a standard pipeline to analyze molecular sequences. Specifically, for the population genetic structure analyses, we used two approaches: visual exploration of the population structure by haplotype networks and the use of the fixation index (Fst), a common measure of population differentiation. Prior to running these methods, we visually inspected the chromatograms (now mentioned in lines 137-138) and performed another visual inspection of the alignments to detect potential amplification or sequencing errors (e.g., internal stop codons).

**Reviewer >>>** Correspondence analysis: It will be interesting to investigate the patterns of distribution based on wing morphology using the relevant high dimensional data reduction method, namely correspondence analysis. It’s a simple analysis based on frequency data to study species distribution based on vestigial and normal wings. Authors already have this data using a huge number of specimens that are reflected in the material examined section under taxonomy.

Trait mapping: As mentioned in the introduction section it will be fascinating to explore the relation between elevation gradient and wing size of Liodessus specimens. Have the authors observed any patterns of reduction in wing size based on elevation gradient? This trait can be further mapped on the phylogeny using Phytools in R.

Have authors also found a mixture of normal-winged and vestigial-winged specimens in the same population?

**REPLY >>>** Thank you for your insight on a potential new analysis regarding wing morphology and adaptation to altitude, which we also are very excited about. Vestigial wings are usually found in high altitudes, as they are a well-known adaptation to cope with extreme conditions such as strong winds. Most specimens in Páramo or Puna ecosystems have vestigial wings. To analyze wing morphology in detail, it would be necessary to incorporate multiple species from various altitudes, not just those inhabiting high altitudes. Unfortunately, we cannot include such an extensive analysis in this manuscript. However, we are working on a broader study with comprehensive sampling and a calibrated phylogenomic analysis of most known *Liodessus* species to answer exactly these questions you asked about speciation and adaptability to high elevations in *Liodessus* diving beetles.

**Reviewer >>>** Line 31: Please change the makred key words. Because these words already appear in the title.

**REPLY >>>** Thank you for the suggestions. We have modified the key words to avoid repetitions with the title.

**Reviewer >>>** Line 89: Please enlist the species and subspecies considered for the current work.

**REPLY >>>** Thank you for your suggestion. That information is included in the revised checklist of the paper

**Updated checklist of *Liodessus* from Colombia and Ecuador**

**(* taxa treated herein)**

*Liodessus bogotensis bogotensis* Guignot, 1953 (Colombia)

*Liodessus b*. *almorzadero* Balke et al., 2023 (Colombia)

*Liodessus b*. *chingaza* Balke et al., 2023 (Colombia)

*Liodessus b*. *lacuniviridis* Balke et al., 2020 (Colombia)

*Liodessus b*. *matarredonda* Balke et al., 2023 (Colombia)

*Liodessus b*. *sumapaz* Balke et al., 2023 (Colombia)

*Liodessus picinus* Balke et al., 2021 (Colombia)

*Liodessus quillacinga quillacinga* Megna, Hendrich & Balke, 2019 (Colombia) *****

*Liodessus quillacinga cochaensis* Megna, Hendrich & Balke, 2019 (Colombia) *****

*Liodessus quillacinga cumbalis* Megna, Hendrich & Balke, 2019 (Colombia) *****

*Liodessus quillacinga ecuadoriensis* ssp. nov. (Ecuador) *****

*Liodessus quimbaya quimbaya* Megna, Hendrich & Balke, 2019 (Colombia) *****

*Liodessus quimbaya azufralis* Megna, Hendrich & Balke, 2019 stat. nov. (Colombia, Ecuador) *****

*Liodessus quimbaya paletara* ssp. nov. (Colombia) *****

*Liodessus riveti* Peschet, 1923 (Ecuador) *****

*Liodessus santarosita Balke et al., 2023 (Colombia)*

**Reviewer >>>** Line 124 and 135: Please elaborate on statistics used.

**REPLY >>>** We have made minor modifications to this section to clarify the methods used. Geneious incorporates a tool to generate neighbor-joining trees using various genetic distance models. More detailed information is provided in the following section (lines 133 to 143).

**Reviewer >>>** Line 149: How the groups of populations were made?

**REPLY >>>** By “population”, we refer to the general definition of individuals of the same species coexisting in the same location and time. As shown in the Fst figures, we maintained the same locality codes as in the manuscript to facilitate comparisons among populations and not only among subspecies, affectively assessing population genetic structure.

**Reviewer >>>** Line 160: Please mention/describe statistical results. Also Line 397.

**REPLY >>>** We agree that mentioning the main statistical results is essential in any scientific manuscript. For that reason, we had included a section on the molecular evidence, where we describe the main results of our analyses.

**Reviewer >>>** Table 1: Are these two populations of the same subspecies? Or subspecies quimbaya is repeated mistakenly?

**REPLY >>>** Thank you for pointing out this issue. It was indeed a repeated word that should not be there. We have corrected the manuscript accordingly.

**Reviewer >>>** Line 453: Has phylogeography of the genus being worked out? Are there any evidences based on the molecular clock about the origin of the genus in such a dynamic ecosystems? Please discuss.

**REPLY >>>** We are currently working on a comprehensive study of *Liodessus*, including most of the described species and some new independent genetic lineages, using whole-genome sequencing and calibrations. However, we are still in the dataset-building phase, and such a work will take some time before it is ready for review. In the meantime, we have been addressing species inconsistencies across *Liodessus* to use that information as a baseline for a comprehensive analysis.

**Reviewer >>>** Line 467-469: Please mention a reference for this sentence or simply remove. Based on the data presented, the current study does not conduct diversity analyes.

**REPLY >>>** The sentence was a continuation of the previous one, referring to the pattern found by Calpe-Anaguano et al. in their study of bird species in the Páramo. We have explicitly incorporated a reference in that sentence to avoid any misunderstanding.

**Reviewer >>>** Reviewer #2: Congratulations on a well written and interesting piece of work, providing deeper taxonomic and biogeographic knowledge on predaceous diving beetles of the northern Andes as study system. Overall subspecies appear to be useful in this taxonomic group, however it seems in some cases they may not be that easy to detect based on morphology, which may pose difficulties for the average entomologist or biomonitor of ecosystems. This may be an intrinsic problem of the group, if some subspecies are in essence cryptic. I recommend in further publications, authors may provide recommendations to biomonitoring professionals and aquatic biologists interested in conservation on how to cope with difficult or cryptic subspecies complexes, either through user-friendly molecular protocols or lower morphological resolution alternatives.

**REPLY >>>** Thank you very much for your kind comments. Indeed, morphological convergence is likely an issue in the *Liodessus* species inhabiting high altitudes in the Andes. To fully understanding how these beetles diversified in such extreme habitats and assess their conservation status, molecular evidence is required. We are aware of the difficulties of dealing with (almost) cryptic species, and we will take your recommendations on biomonitoring and conservation into account for future studies.

**Reviewer >>>** Line 59-60:

**REPLY >>>** We did not specify a country because Páramo and Puna are the names of high-altitude plateaus in the Andes, with Páramo most frequently used in northern Andes and Puna in Central and Southern Andes.

**Reviewer >>>** Line 262: is there any additional distinguishing feature from geographic locality?

**REPLY >>>** No. We intend to highlight that the subspecies is characterized by its geographic location.
